# The ubiquitin E3 ligase TRAF6 exacerbates pathological cardiac hypertrophy via TAK1-dependent signalling

**DOI:** 10.1038/ncomms11267

**Published:** 2016-06-01

**Authors:** Yan-Xiao Ji, Peng Zhang, Xiao-Jing Zhang, Yi-Chao Zhao, Ke-Qiong Deng, Xi Jiang, Pi-Xiao Wang, Zan Huang, Hongliang Li

**Affiliations:** 1 Department of Cardiology, Renmin Hospital of Wuhan University, Wuhan 430060, China; 2 Animal Experiment Center/Animal Biosafety Level-III Laboratory, Wuhan University, Wuhan 430060, China; 3 Medical Research Institute, School of Medicine, Wuhan University, Wuhan 430071, China; 4 Department of Cardiology, Shanghai Renji Hospital, School of Medicine, Shanghai Jiaotong University, Shanghai 200127, China; 5 College of Life Science, Wuhan University, Wuhan 430072, China

## Abstract

Tumour necrosis factor receptor-associated factor 6 (TRAF6) is a ubiquitin E3 ligase that regulates important biological processes. However, the role of TRAF6 in cardiac hypertrophy remains unknown. Here, we show that TRAF6 levels are increased in human and murine hypertrophied hearts, which is regulated by reactive oxygen species (ROS) production. Cardiac-specific *Traf6* overexpression exacerbates cardiac hypertrophy in response to pressure overload or angiotensin II (Ang II) challenge, whereas Traf6 deficiency causes an alleviated hypertrophic phenotype in mice. Mechanistically, we show that ROS, generated during hypertrophic progression, triggers TRAF6 auto-ubiquitination that facilitates recruitment of TAB2 and its binding to transforming growth factor beta-activated kinase 1 (TAK1), which, in turn, enables the direct TRAF6–TAK1 interaction and promotes TAK1 ubiquitination. The binding of TRAF6 to TAK1 and the induction of TAK1 ubiquitination and activation are indispensable for TRAF6-regulated cardiac remodelling. Taken together, we define TRAF6 as an essential molecular switch leading to cardiac hypertrophy in a TAK1-dependent manner.

## Abstract (web summary)

TRAF6 is a ubiquitin E3 ligase regulating a number of biological processes. Here the authors show that ROS, generated during pathological cardiac stress, induces TRAF6 auto-ubiquitination and activation, promoting its interaction with and ubiquitination of TAK1 that contributes to development of cardiac hypertrophy.

## Graphical abstract

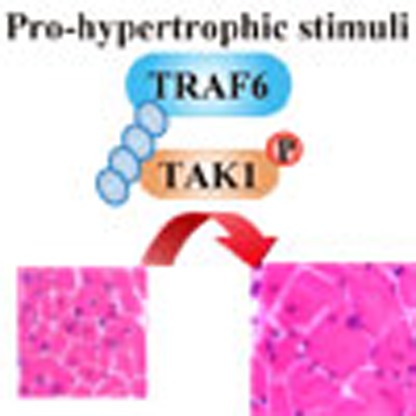

Pathological cardiac hypertrophy, which is characterized by myocyte enlargement and dysfunctional cardiac contractility, is a major predisposing factor for heart failure, arrhythmia and sudden death^1^. In recent decades, intensive investigations of pathological cardiac hypertrophy have promoted the identification of a series of cellular pathologies. Generally, cardiac hypertrophic stimuli result in the excessive activation of a complex signalling cascade, for example, the mitogen-activated protein kinase (MAPK), PI3K/Akt, NF-κB and calcineurin/nuclear factor of activated T cells pathways, which converge on corresponding transcription factors and lead to abnormal protein synthesis and pathological cardiac remodelling^2^,^3^. Despite accumulating evidence, the ubiquitous roles of these pathways in cell biology make the systemic and off-target effects of direct manipulation a major hurdle to successful clinical translation. Therefore, a better understanding of the pathogenesis of cardiac hypertrophy and the identification of novel therapeutic targets are greatly needed.

The tumour necrosis factor receptor (TNFR)-associated factor (TRAF) family members (TRAF1–7 in mammals), characterized by a C-terminal TRAF domain (except TRAF7) and N-terminal RING finger motif (except TRAF1), are important adaptor proteins with enzymatic activities. They play indispensable roles in regulating innate and adaptive immunity, embryonic development, tissue homoeostasis and bone metabolism^4^,^5^. Recently, our group and others have explored the therapeutic potential of TRAF2/3/5 for cardiac hypertrophy^6^,^7^,^8^, suggesting a possible involvement of the TRAF family in pathological cardiac remodelling. Among the TRAF members, TRAF6 possesses non-conventional ubiquitin E3 ligase activity similar to that of TRAF2/3/5 and plays non-redundant functions in tissue homoeostasis and immune response^4^,^9^,^10^. Diverse TRAF6-interacting motifs on its binding partners provide TRAF6 with a broad spectrum of substrates, including membrane receptors, intracellular kinases and adaptor proteins^11^,^12^,^13^. Notably, TRAF6 exclusively exhibits a direct regulatory effect on transcription factors such as IRF7 (refs 14, 15), which are distal convergence nodes of various hypertrophic signalling pathways^16^. In addition, *Traf6* is prominently expressed in the heart^17^, and cardiac levels of TRAF6 tend to be upregulated in endotoxin-induced cardiomyopathy and CVB3-induced myocarditis^18^,^19^. These data suggest a potential functional involvement of TRAF6 in cardiac pathology, especially in terms of cardiac hypertrophy. However, supporting data for this hypothesis is largely lacking.

In the present study, we observe elevated protein levels of TRAF6 in the hearts of patients with hypertrophic cardiomyopathy (HCM) or dilated cardiomyopathy (DCM) and in animal models of cardiac hypertrophy induced by aortic banding (AB). Using gain- and loss-of-function approaches *in vivo* and *in vitro*, we demonstrate that TRAF6 polyubiquitination and activation are induced by reactive oxygen species (ROS) production during hypertrophic progression, and the activated TRAF6 aggravates the progression of pathological cardiac remodelling by interacting with and promoting the ubiquitination of transforming growth factor beta-activated kinase 1 (TAK1), followed by the activation of the TAK1-JNK/p38 axis.

## Results

### TRAF6 expression is increased in hypertrophic hearts

**Figure 1:**
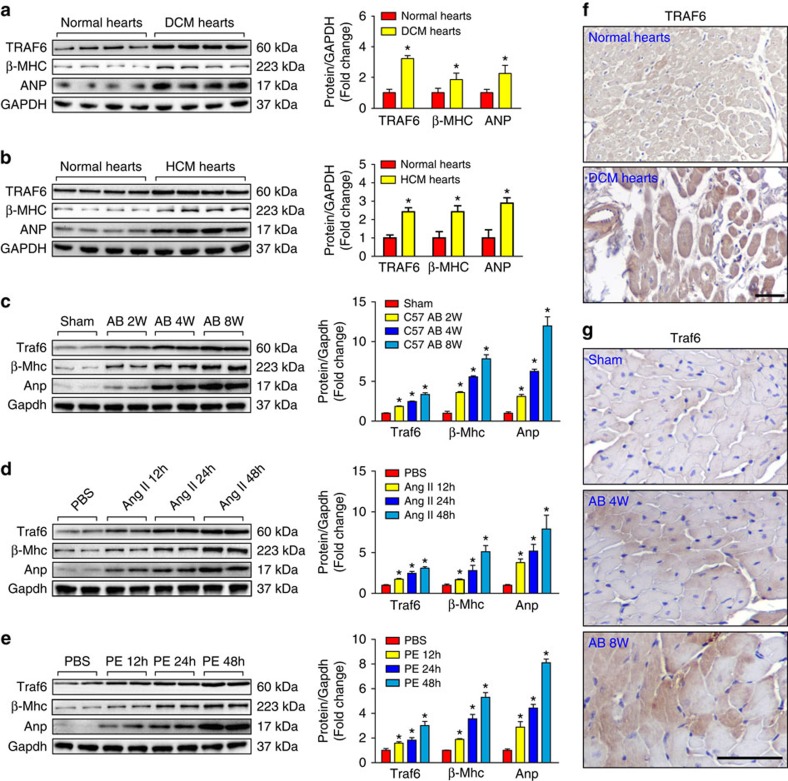
TRAF6 expression is upregulated by hypertrophic stimuli. (**a**,**b**) Western blot analysis and quantitative results of TRAF6, β-myosin heavy chain (β-MHC), and atrial natriuretic peptide (ANP) levels in human heart samples from normal donors and patients with dilated cardiomyopathy (DCM) (**a**) or hypertrophic cardiomyopathy (HCM) (**b**) (*n*=6 for normal heart group, *n*=7 for DCM group, *n*=5 for HCM group, *P<0.05 versus normal hearts). (**c**) Western blot analysis and quantitative results of Traf6, β-Mhc and Anp in a murine model of cardiac hypertrophy induced by aortic banding (AB) at the indicated time points (*n*=4 mice/group, *P<0.05 versus sham). (**d**,**e**) The expression of Traf6, β-MHC and ANP in neonatal rat cardiomyocytes (NRCMs) treated with PBS, angiotensin II (Ang II, 1 μM) (**d**) or phenylephrine (PE, 50 μM) (**e**) (*P<0.05 versus PBS). Protein expression levels were normalized to GAPDH for western blotting in **a**–**e**. (**f**,**g**) Representative images of immunohistochemistry with an anti-TRAF6 antibody in slices from the indicated human heart (**f**) or mouse heart (**g**) samples (*n*=5/group; scale bar, 50 μm). Data are presented as the mean±s.d. from at least three independent experiments. For **a** and **b** statistical analysis was carried out by Student's two-tailed *t*-test; for **c**–**e** statistical analysis was carried out by one-way ANOVA.

To explore the potential involvement of TRAF6 in the development of pathological cardiac hypertrophy, we first investigated whether TRAF6 expression was altered in failing human hearts and in a mouse model of cardiac hypertrophy. Western blotting results indicated that TRAF6 protein levels were significantly increased in DCM and HCM human hearts (Fig. 1a,b), which occurred in parallel with the upregulation of fetal genes, including atrial natriuretic peptide (ANP) and β-myosin heavy chain (β-MHC; Fig. 1a,b). In experimental hypertrophic models, TRAF6 protein levels were progressively elevated in mouse hearts from 2 to 8 weeks after AB surgery compared with sham-operated controls (1.8, 2.5 and 3.4-fold at 2, 4 and 8 weeks, respectively) (Fig. 1c). In addition, TRAF6 upregulation was found in isolated neonatal rat cardiomyocytes (NRCMs) that were stimulated with angiotensin II (Ang II) or phenylephrine (PE; Fig. 1d,e) for 12, 24 or 48 h compared with phosphate-buffered saline (PBS)-treated controls. More importantly, the augmented *TRAF6/Traf6* expression in hypertrophic heart tissues was mainly localized to cardiomyocytes, as demonstrated by immunohistochemistry in human and mouse heart sections (Fig. 1f,g). Taken together, the increased *TRAF6/Traf6* expression in heart samples with cardiac remodelling suggests that TRAF6 may be implicated in the pathogenesis of cardiac hypertrophy.

### *Traf6* expression is increased by ROS production

Next, we investigated the reason for the significant TRAF6 increase during cardiac remodelling. Among the numerous pro-hypertrophic factors, ROS has been highlighted for its crucial participation in various cardiovascular diseases (for example, hypertension, atherosclerosis and heart failure)^20^,^21^. More importantly, intracellular ROS is required for the activation of TRAF6 (refs 22, 23, 24). Thus, the ROS contents and the activation of their major source, NADPH oxidases, were measured. As shown in Supplementary Fig. 1a, the ROS production level was significantly increased in heart sections from mice 2 or 4 weeks after AB surgery compared with those of sham controls. Consistently, a dramatic increase in ROS contents and NADPH oxidase activity in the heart was observed after 4 weeks of pressure overload challenge compared with the control group and was almost completely abolished by the administration of the NADPH oxidase inhibitor apocynin (APO) or the ROS scavenger N-acetyl-cysteine (NAC) (Supplementary Fig. 1b and c). In addition, the activities of antioxidant enzymes were examined. As shown in Supplementary Fig. 1d–f, the enzymatic activities of glutathione peroxidase (GPx), catalase (CAT) and superoxide dismutase (SOD) were all dramatically reduced by AB surgery but were nullified by NAC treatment. In addition to those observed in AB-treated mice, consistent phenotypes regarding ROS generation were observed in mice subjected to Ang II infusion for 28 days compared with control mice treated with saline (Supplementary Fig. 1b–f). Furthermore, the elevation of intracellular ROS production and the inhibition of ROS production by NAC and APO were validated in primary NRCMs stimulated with Ang II for 24 h (Supplementary Fig. 1g).

**Figure 2:**
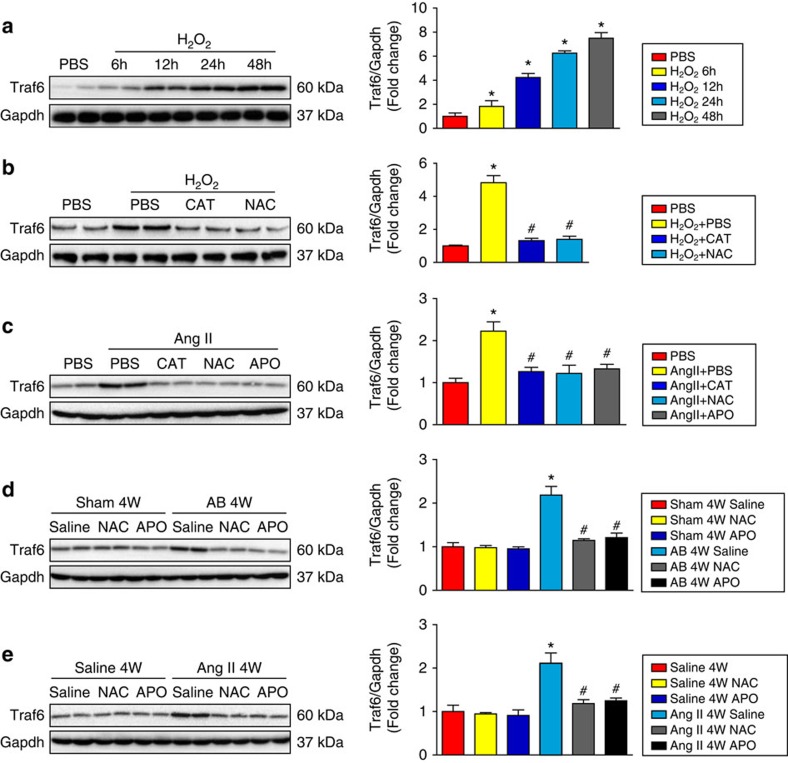
ROS production regulates Traf6 expression during cardiac hypertrophy. (**a**,**b**) The protein expression of Traf6 in NRCMs treated with H_2_O_2_ for 6, 12, 24 or 48 h (**a**) and in cardiomyocytes treated for 24 h with or without CAT or NAC administration (**b**). (*P<0.05 versus PBS, ^#^*P*<0.05 versus H_2_O_2_+PBS group). (**c**) The expression level of Traf6 in the NRCMs after 24 h of Ang II administration with or without CAT, NAC or APO treatment. (*P<0.05 versus PBS, ^#^*P*<0.05 versus Ang II+PBS). (**d**,**e**) Traf6 protein expression in heart samples of AB− (**d**) or Ang II-treated (**e**) mice after 4 weeks of pro-hypertrophic stimuli. (*n*=4 mice per experimental group, in **d** *P<0.05 versus Sham+saline group, ^#^*P*<0.05 versus AB+saline group; in **e** *P<0.05 versus Saline group, ^#^*P*<0.05 versus Ang II+saline group). Gapdh served as a loading control. Data are presented as the mean±s.d. from at least three independent experiments. Statistical analysis was carried out by one-way ANOVA.

To evaluate whether ROS production contributes to increased *TRAF6* expression during cardiac hypertrophy, we subjected NRCMs to H_2_O_2_ stimulus and found that TRAF6 protein expression was gradually enhanced from 6 to 48 h of H_2_O_2_ challenge (Fig. 2a,b). As expected, Ang II treatment led to a dramatic elevation of Traf6 expression in primary cardiomyocytes (Fig. 2c), and increased expression levels of Traf6 were also found in the heart upon AB surgery or Ang II infusion *in vivo* (Fig. 2d,e). Administration of CAT, NAC or APO largely reversed the increased Traf6 expression induced by pro-hypertrophic stimuli (Fig. 2b–e). Collectively, these data demonstrate that the activation of NADPH oxidase and the production of ROS during cardiac remodelling are largely responsible for the induction of Traf6 expression.

### Traf6 aggravates cardiac hypertrophy under pressure overload

**Figure 3:**
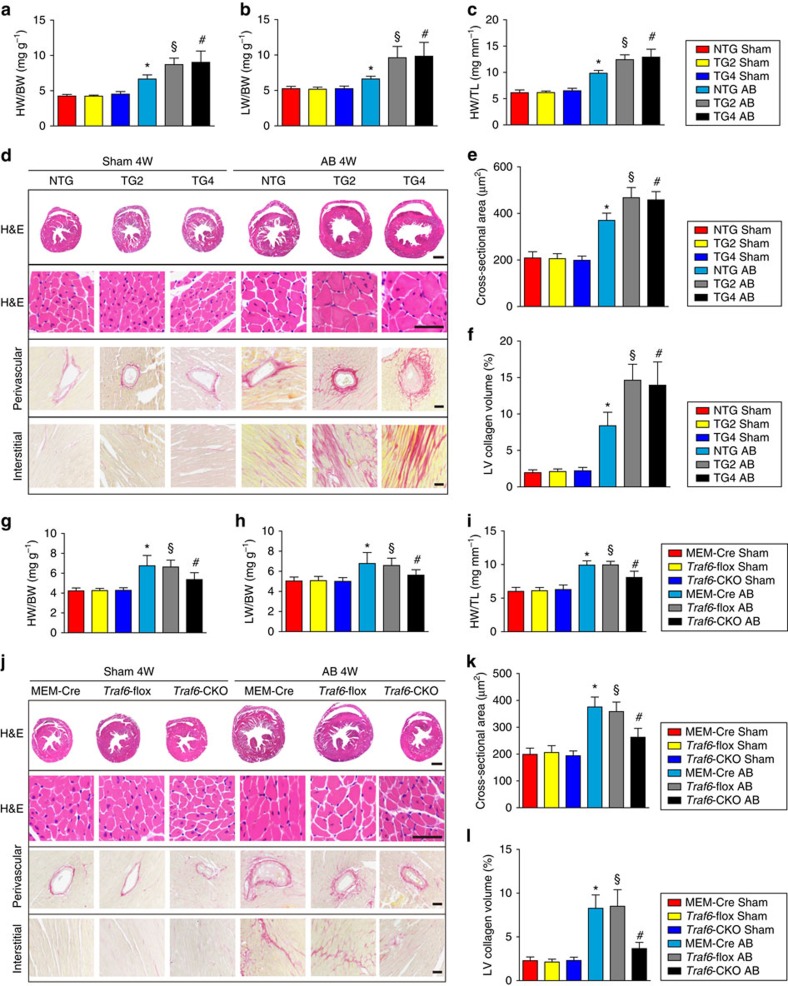
Traf6 promotes AB-induced cardiac hypertrophy. (**a**–**c**) The ratios of heart weight to body weight (HW/BW) (**a**) lung weight to body weight (LW/BW) (**b**) and heart weight to tibia length (HW/TL) (**c**) were determined in the indicated groups 4 weeks after AB surgery (*n*=11–13 mice per group). (**d**) Histological analysis of heart slices by HE staining and PSR staining to assess cardiomyocyte cross-sectional areas and cardiac fibrosis, respectively, 4 weeks after AB surgery (*n*=6–8 mice/group; scale bar, 1,000 μm for the upper most set of panels and scale bar, 50 μm for lower panels). (**e**) Statistical results for the cell cross-sectional area (*n*>100 cells per group). (**f**) Statistical results for the fibrotic areas in the indicated groups (*n*>40 fields per group). (**g**–**i**) The HW/BW (**g**) LW/BW (**h**) and HW/TL (**i**) were assessed in the indicated groups following 4 weeks of AB treatment (*n*=11–12 mice per group). (**j**) Sections of hearts from the indicated groups were stained with HE and PSR to analyse heart and cardiomyocyte size and fibrotic area, respectively (*n*=6–8 mice/group; scale bar, 1,000 μm for the upper most set of panels and scale bar, 50 μm for lower panels). (**k**) Statistical results for the cross-sectional area of cardiomyocytes in different groups (*n*>100 cells per group). (**l**) Statistical results for the fibrotic areas in the indicated groups (*n*>40 fields/group). For (**a**–**f**) *P<0.05 versus NTG sham; ^§^*P*<0.05 versus NTG AB, ^#^*P*<0.05 versus NTG AB. For **g**–**l**, *P<0.05 versus MEM-Cre sham; ^§^*P*<0.05 versus *Traf6*-flox sham, ^#^*P*<0.05 versus *Traf6*-flox AB. Data are presented as the mean±s.d. Statistical analysis was carried out by one-way ANOVA.

The dramatic increase in Traf6 induced by ROS production upon pro-hypertrophic administration prompted us to investigate whether the increased Traf6 contributes to cardiac hypertrophy. We therefore generated mice with cardiac-specific overexpression of *Traf6* (TG) to evaluate the function of Traf6 in hypertrophied hearts. The generation of TG mice was derived by the cardiac-specific α-myosin heavy chain (*α-MHC*) promoter (Supplementary Fig. 2a). Four *Traf6*-TG mouse lines were generated, and *Traf6* overexpression in the heart was validated by western blotting (Supplementary Fig. 2b). The TG2 and TG4 lines were then randomly selected for further experiments. In response to pressure overload, *Traf6* overexpression significantly aggravated cardiac hypertrophy, as evidenced by increased ratios of heart weight (HW) to body weight (BW), lung weight (LW) to BW and HW to tibial length (TL; Fig. 3a–c). Furthermore, the *Traf6*-TG mice exhibited markedly elevated left ventricle end-diastolic dimension (LVEDd) and left ventricle end-systolic dimension (LVESd) and decreased fractional shortening (FS) values compared with the non-transgenic (NTG) controls (Supplementary Fig. 2c). In addition, hematoxylin-eosin (HE) or picrosirius red (PSR) staining of heart sections indicated that the hearts of *Traf6*-TG mice developed significantly increased cardiomyocyte hypertrophy and fibrosis in the interstitial and perivascular spaces compared with their NTG littermates (Fig. 3d–f). Consistently, the mRNA levels of markers related to cardiac hypertrophy and fibrosis, including *Anp*, *Bnp*, *β-Mhc*, collagen I, collagen III and *Ctgf*, were markedly elevated in the hearts of *Traf6*-TG mice compared with NTG controls (Supplementary Fig. 2d). Collectively, these results indicate that the upregulated Traf6 levels in the heart promote pressure overload-induced cardiac hypertrophy and the resultant heart dysfunction.

In addition to the *Traf6*-TG mice, a cardiac-specific *Traf6* deletion mouse line (*Traf6*-CKO) was created and subjected to AB surgery to further confirm the regulatory function of Traf6 in cardiac hypertrophy. A *Traf6*-flox-targeted allele (*Traf6*-flox) and a cardiac-specific *α-Mhc*-MerCreMer transgenic line (MEM-Cre) served as control littermates. Western blot analysis demonstrated a specific Traf6 deletion in the hearts of *Traf6*-CKO mice (Supplementary Fig. 2e). Four weeks after AB surgery, *Traf6*-CKO mice exhibited a notable alleviation of cardiac enlargement and cardiac dysfunction compared with the *Traf6*-flox and MEM-Cre controls, as indicated by decreased HW/BW, LW/BW and HW/TL ratios (Fig. 3g–i) and ameliorated cardiac dysfunction (Supplementary Fig. 2f). Histologic analysis further revealed that AB-induced cardiomyocyte hypertrophy and interstitial and perivascular fibrosis were drastically attenuated in the *Traf6*-CKO mice (Fig. 3j–l). Correspondingly, the transcription levels of hypertrophic and fibrotic markers were significantly suppressed in the CKO mice compared with heart samples from the *Traf6*-flox or MEM-Cre controls (Supplementary Fig. 2g). Interestingly, neither Traf6 overexpression nor ablation significantly influenced the cardiac morphological or functional characteristics under basal conditions compared with their littermate controls. Taken together, these *in vivo* gain- and loss-of-function studies demonstrate that elevated cardiac Traf6 expression is essential for the development of the pathological cardiac hypertrophy induced by chronic pressure overload.

### Traf6 promotes cardiac hypertrophy in response to Ang II

In addition to chronic pressure overload, neurohormonal stimuli (for example, Ang II) are common triggers resulting in cardiac hypertrophy^2^,^25^. To exclude the possibility that these effects of Traf6 were specific to pressure overload-induced cardiac hypertrophy, we next investigated whether Traf6 exacerbated Ang II-induced cardiac hypertrophy *in vivo.* As shown in Supplementary Fig. 3, 4 weeks of Ang II infusion in wild-type (WT) mice led to significantly increased HW/BW, LW/BW and HW/TL ratios compared with saline controls, as well as impaired contractile function and enlarged myocytes. The structural and functional remodelling of the heart induced by Ang II infusion were significantly exacerbated by Traf6 overexpression (Supplementary Fig. 3a–f), whereas *Traf6*-CKO mice displayed markedly alleviated cardiac hypertrophy and dysfunction (Supplementary Fig. 3g–l). These data indicate that Traf6 represents an essential mediator of the pathological cardiac hypertrophy induced by both pressure overload and hormonal stresses.

### Traf6 promotes cardiomyocytes hypertrophy *in vitro*

**Figure 4:**
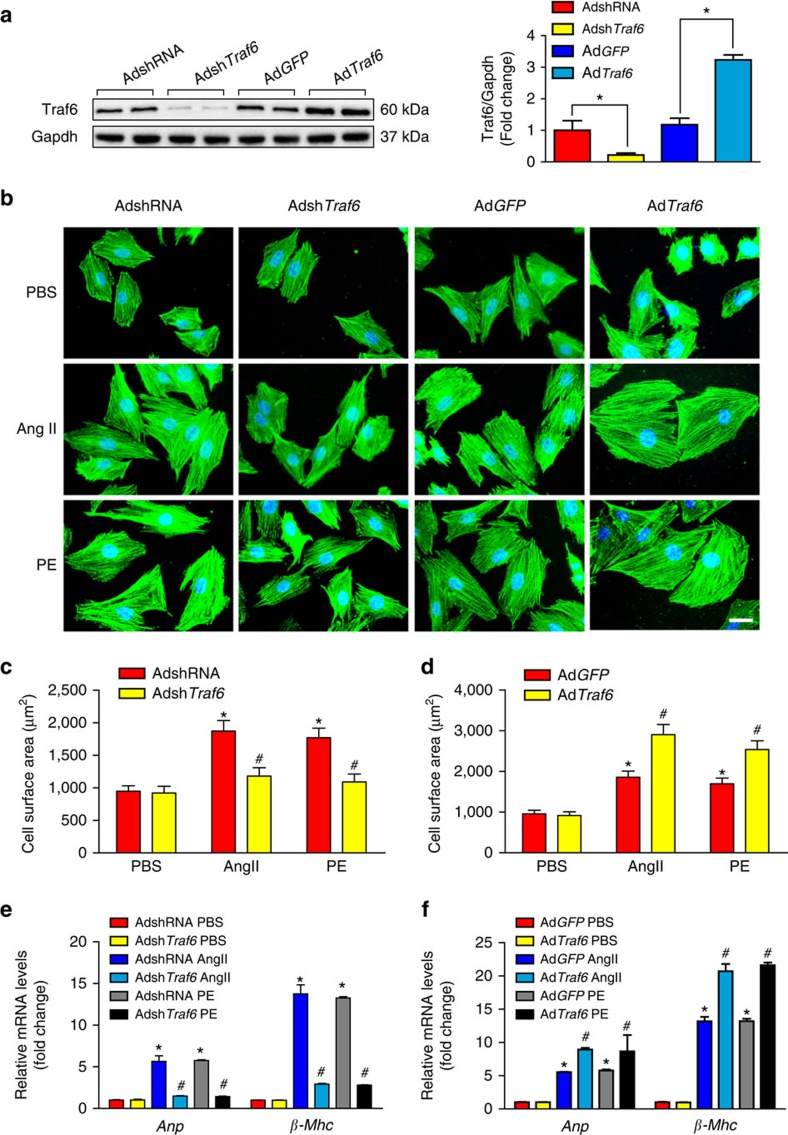
Traf6 promotes Ang II/PE-induced cardiomyocyte hypertrophy *in vitro*. (**a**) Primary cardiomyocytes were infected with AdshRNA, Adsh*Traf6*, Ad*GFP* or Ad*Traf6* for 24 h. Cells were then harvested, and cellular lysates were analysed by western blotting to measure Traf6 expression levels (*P<0.05 versus AdshRNA or Ad*GFP*). (**b**) Representative microscopic images of cardiomyocytes infected with the indicated adenoviruses and treated with PBS, Ang II or PE for 48 h. Cardiomyocytes were identified by α-actinin staining (green), and nuclei were stained with DAPI (blue). Scale bars, 20 μm. (**c**,**d**) Quantitative results of the cell surface area of cardiomyocytes infected with Adsh*Traf6* or Ad*Traf6* as well as their AdshRNA or Ad*GFP* controls in response to PBS, Ang II or PE for 48 h (*n*>50 cells/group, *P<0.05 versus AdshRNA PBS or Ad*GFP* PBS, ^#^*P*<0.05 versus AdshRNA Ang II/PE or Ad*GFP* Ang II/PE). (**e**,**f**) Real-time quantitative PCR was performed to determine the transcriptional levels of *Anp* and *β-Mhc* in adenovirus-infected cardiomyocytes after treatment with PBS, Ang II or PE for 48 h (*P<0.05 versus AdshRNA PBS or Ad*GFP* PBS, ^#^*P*<0.05 versus AdshRNA Ang II/PE or Ad*GFP* Ang II/PE). Data are presented as the mean±s.d. from at least three independent experiments. Statistical analysis was carried out by one-way ANOVA.

Because cardiomyocyte enlargement is the defining characteristic of cardiac remodelling, we further evaluated the specific role of Traf6 in cardiomyocytes by infecting NRCMs with adenovirus harbouring *Traf6* short hairpin RNA (Adsh*Traf6*) or *Traf6* cDNA (Ad*Traf6*) (Fig. 4a). Myocyte enlargement was induced by Ang II or PE administration. The cellular surface areas of cardiomyocytes were measured by immunostaining with α-actinin 48 h after Ang II or PE treatment. Ad*Traf6* notably augmented the Ang II/PE-induced cardiomyocyte enlargement compared with Ad*GFP* transfection, accompanied by significant increases in the fetal genes *Anp* and *β-Mhc*, whereas *Traf6* silencing significantly alleviated myocyte hypertrophy and downregulated the expression of *Anp* and *β-Mhc* compared with primary cells infected with AdshRNA (Fig. 4b–f), suggesting that Traf6 can directly promote the hypertrophic growth of isolated myocytes induced by Ang II or PE. The combined *in vivo* and *in vitro* experiments clearly validated an essential role for Traf6 during the initiation and progression of pathological cardiac hypertrophy.

### ROS mediates the effect of Traf6 on cardiac remodelling

**Figure 5:**
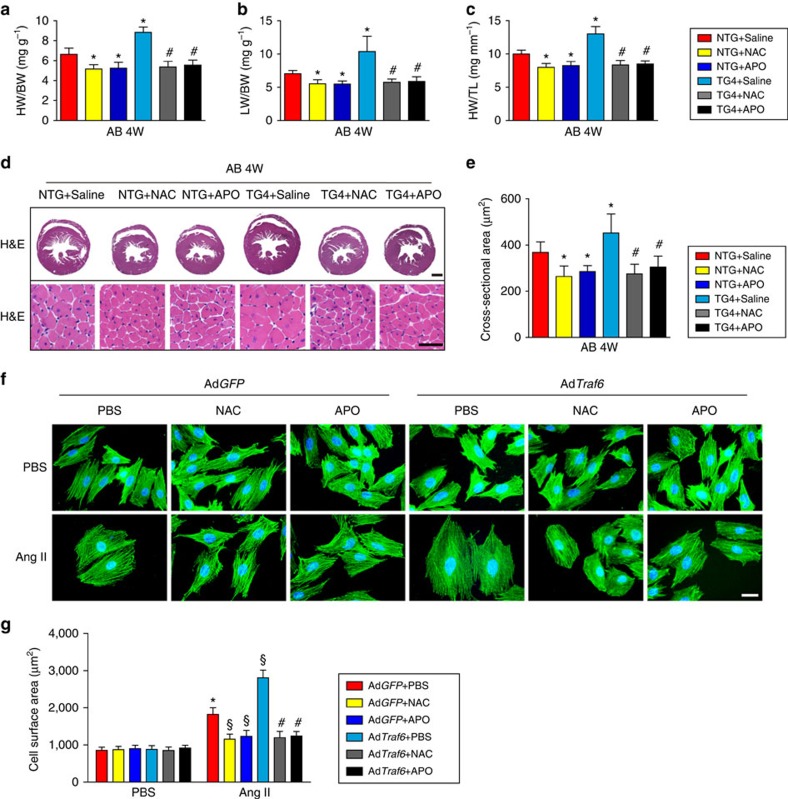
Blocking ROS reverses Traf6-regulated exacerbation of cardiac remodeling. (**a**–**c**) The HW/BW (**a**) LW/BW (**b**) and HW/TL (**c**) ratios in the *Traf6*-TG4 mice and their NTG controls treated with saline, NAC or APO after AB surgery for 4 weeks (*n*=11–13 per group in **a**–**c**). (**d**,**e**) Representative images of HE staining (*n*=6–8 mice per group; scale bar, 1,000 μm for the upper most set of panels and scale bar, 50 μm for lower panels, (**d**)) and the cross-sectional areas (*n*>100 cells per group, (**e**)) of heart sections from mice in the indicated groups. In **a**–**e** *P<0.05 versus NTG saline group; ^#^*P*<0.05 compared to TG4 saline group. (**f**,**g**) Representative images (scale bar, 20 μm, (**f**)) and calculated cell surface areas (*n*>50 cells per group, (**g**)) of α-actinin-stained NRCMs that were infected with Ad*GFP* or Ad*Traf6* and treated with PBS or Ang II in the presence or absence of NAC or APO. *P<0.05 versus Ad*GFP*/PBS+PBS group; ^§^*P*<0.05 versus Ad*GFP*/Ang II+PBS group; ^#^*P*<0.05 versus Ad*Traf6*/Ang II+PBS group. Data are presented as the mean±s.d. Statistical analysis was carried out by one-way ANOVA.

Based on the fact that ROS production induces a dramatic increase in Traf6 expression that significantly promotes cardiac hypertrophy development, we next asked whether ROS blockage could neutralize the pro-hypertrophic effect of Traf6. To address this question, we introduced an ROS scavenger (NAC) or NADPH oxidase inhibitor (APO) into the NTG and *Traf6*-TG mice, followed by a continuous pressure overload challenge for 4 weeks. As expected, blocking ROS production with NAC or APO markedly reversed the cardiac hypertrophy and dysfunction induced by AB surgery in both NTG and *Traf6*-TG mice, as evidenced by their decreased HW/BW (Fig. 5a), LW/BW (Fig. 5b) and HW/TL ratios (Fig. 5c); normalized LVEDd, LVESd and FS values (Supplementary Fig. 4a); and reduced cross-sectional area visualized by HE staining (Fig. 5d,e) compared with mice treated with saline administration. Moreover, the addition of ROS scavenger or NADPH oxidase inhibitor largely abolished the TRAF6-induced increase in cardiomyocyte enlargement and elevation of *Anp* and *β-Mhc* mRNA levels (Fig. 5f–g; Supplementary Fig. 4b). Therefore, it could be reasonably deduced that ROS production is required for the activation of Traf6 and its regulation of cardiac remodelling.

### TRAF6 regulates cardiac hypertrophy via TAK1-JNK1/2/p38 axis

**Figure 6:**
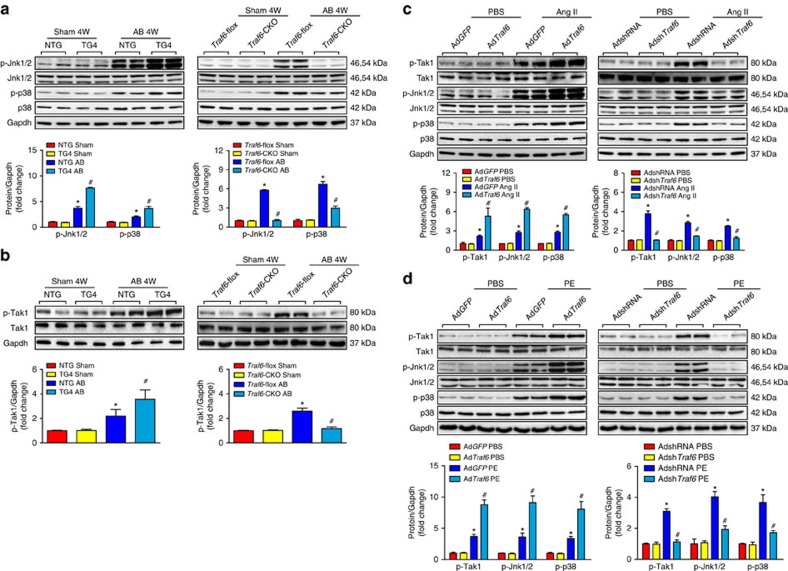
The TAK1-JNK1/2/p38 axis is involved in TRAF6-regulated cardiac hypertrophy. (**a**) Western blots showing the phosphorylation and total protein levels of Jnk1/2 and p38 in heart tissues from NTG and TG4 mice (left) or WT and *Traf6*-CKO mice (right) 4 weeks after AB surgery. (**b**) The phosphorylation and total protein levels of Tak1 in heart tissues in the indicated groups. For (**a**,**b**) *n*=4 mice per group, *P<0.05 versus *Traf6*-flox sham or NTG sham; ^#^*P*<0.05 versus *Traf6*-flox AB or NTG AB. (**c**,**d**) The protein levels of phosphorylated and total Tak1, Jnk1/2 and p38 in NRCMs infected with Ad*GFP* and Ad*Traf6* (left) or AdshRNA and Adsh*Traf6* (right) treated with Ang II (**c**) or PE (**d**) (*P<0.05 versus Ad*GFP* PBS or AdshRNA PBS, ^#^*P*<0.05 versus Ad*GFP* Ang II/PE or AdshRNA Ang II/PE). Data are presented as the mean±s.d. from at least three independent experiments. Statistical analysis was carried out by one-way ANOVA.

Our functional studies provided robust evidence that the increased Traf6 expression induced by ROS production positively regulates the development of pathological cardiac hypertrophy. To gain insight into the molecular mechanisms underlying the effects of TRAF6, we examined the downstream pathways involved in the TRAF6-mediated hypertrophic response. Given the capacity of TRAF6 to regulate AKT activation and the participation of AKT signalling in pathological cardiac hypertrophy^26^,^27^, we first evaluated the potential involvement of AKT signalling in the pro-hypertrophic effect of Traf6. However, despite the significantly elevated levels of Akt phosphorylation induced by hypertrophic stress, we observed no significant alterations in Akt phosphorylation levels by either Traf6 overexpression or depletion (Supplementary Fig. 5a). We then investigated the potential regulatory effects of Traf6 on MAPK signalling pathways, which are well-established pro-hypertrophic kinase cascades that comprise three subfamilies: the Jnk1/2, Erk1/2 and p38 cascades. Western blotting results demonstrated that, compared with NTG controls, Traf6 overexpression selectively activated Jnk1/2 and p38 signalling, but not Mek1/2-Erk1/2 signalling, in response to AB surgery (Fig. 6a; Supplementary Fig. 5a). Accordingly, Jnk1/2 and p38 phosphorylation levels were markedly decreased in hypertrophic hearts in *Traf6*-CKO mice compared with *Traf6*-flox controls (Fig. 6a; Supplementary Fig. 5a).

**Figure 7:**
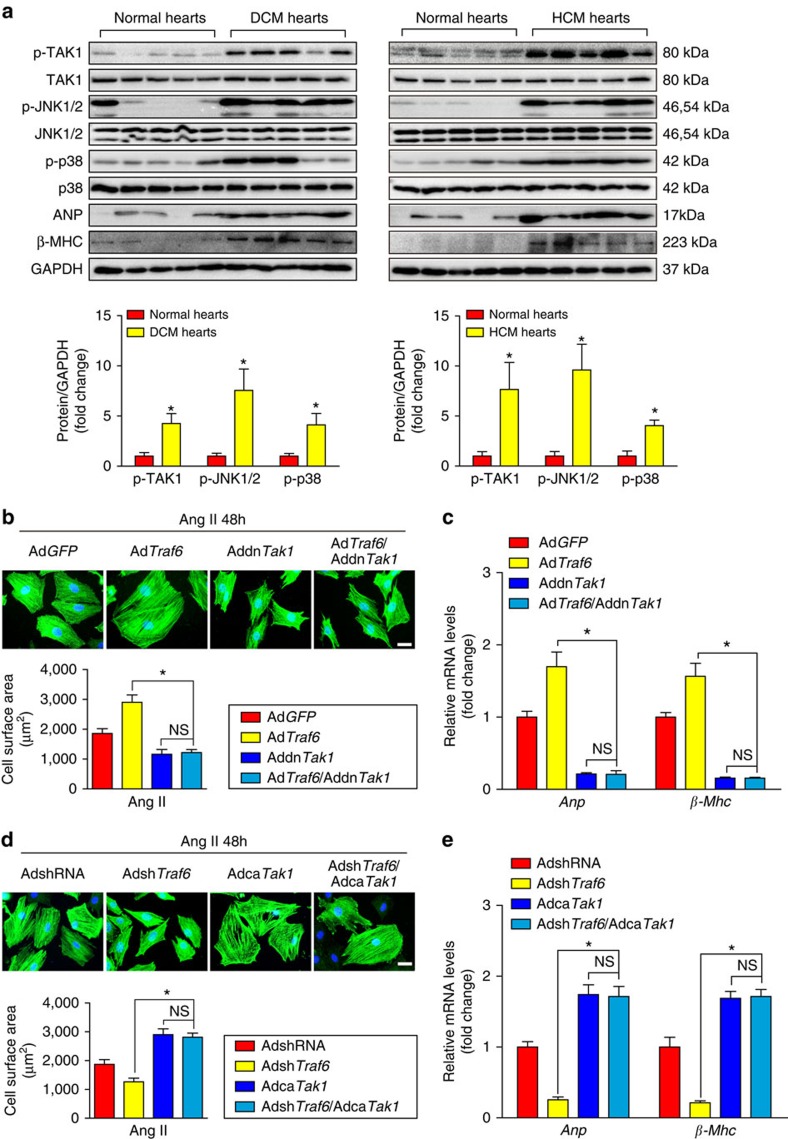
TRAF6 mediates cardiac hypertrophy dependent on TAK1-JNK1/2/p38 signaling. (**a**) The protein expression levels of p-TAK1, TAK1, p-JNK1/2, JNK1/2, p-p38, p38, ANP and β-MHC in heart tissues from normal donors, DCM patients (left), or HCM patients (right) (*n*=5–7/group, *P<0.05 versus normal hearts). (**b**) Representative images and quantified size of cardiomyocytes stained with the α-actinin antibody. (*n*>50 cells per group, *P<0.05 versus Ad*Traf6*, NS, not significant). (**c**) The mRNA expression levels of *Anp* and *β-Mhc* in indicated adenovirus-infected cardiomyocytes after treatment with Ang II for 48 h (*n*=4 per group, *P<0.05 versus Ad*Traf6*, NS, no significance). (**d**) Representative images and quantified cross-sectional areas of cardiomyocytes in the indicated groups stained with the α-actinin antibody (*n*>50 cells per group, *P<0.05 versus Adsh*Traf6*, NS, no significance). (**e**) The mRNA levels of *Anp* and *β-Mhc* of cardiomyocytes in the indicated groups after treatment with Ang II for 48 h (*n*=4 per group, *P<0.05 versus Adsh*Traf6*, NS, no significance). Protein expression levels were normalized to GAPDH. Data are presented as the mean±s.d. from at least three independent experiments. For (**a**) statistical analysis was carried out by Student's two-tailed *t*-test; for (**b**–**e**) statistical analysis was carried out by one-way ANOVA.

JNK1/2 and p38 are regulated by several upstream kinases in response to various stresses^28^,^29^. To further investigate the underlying mechanism by which TRAF6 regulates JNK1/2 and p38 phosphorylation, we examined the regulatory effects of TRAF6 on potential kinases upstream of JNK1/2 and p38. Analysis of the upstream kinases revealed that Tak1 activation was selectively augmented by Traf6 overexpression but suppressed by Traf6 ablation (Fig. 6b), whereas negligible effects were observed on the phosphorylation levels of Tbk1, Ask1, Pi3k, Ilk and Fak in response to hypertrophic stresses (Supplementary Fig. 5b). Consistent with the *in vivo* results, adenovirus-mediated Traf6 expression in isolated NRCMs exposed to Ang II corroborated the positive roles of Traf6 in Tak1 phosphorylation and in Jnk1/2 and p38 activation (Fig. 6c), instead of the other potential signalling cascades examined in the present study (Supplementary Fig. 5c,d). Furthermore, we observed that Traf6 was essential for the activation of Tak1-Jnk1/2/p38 signalling in enlarged NRCMs induced by PE, another important pro-hypertrophic stimulus (Fig. 6d). Importantly, we observed significantly elevated TAK1, JNK1/2 and p38 phosphorylation levels, along with increased ANP and β-MHC, in heart samples from DCM and HCM patients compared with those of normal controls, indicating a close association between TRAF6 and the TAK1-JNK1/2/p38 axis during the clinical development of cardiac remodelling (Fig. 7a). Collectively, the results indicate that the pro-hypertrophic effect of TRAF6 is strongly associated with the activation of TAK1-JNK1/2/p38 signalling.

To further illuminate whether the activation of TAK1–JNK1/2/p38 signalling is required for TRAF6-mediated cardiac hypertrophy, we co-transfected NRCMs with Ad*Traf6* and Addn*Tak1*, which carries a dominant-negative Tak1 mutant, or co-transfected NRCMs with Adsh*Traf6* and Adca*Tak1*, which carries a constitutively activating Tak1 mutant. We then subjected the NRCMs to Ang II treatment. As shown in Fig. 7b,c, Addn*Tak1* transfection triggered a dramatic amelioration of cardiomyocyte enlargement and largely reversed the pathological effects of Traf6 overexpression. In contrast, Adca*Tak1* significantly diminished the protective effect of Traf6 knockdown, leading to a hypertrophic phenotype comparable to that of NRCMs overexpressing Adca*Tak1* alone (Fig. 7d,e). Together, these results demonstrate that Traf6 promotes myocyte hypertrophy dependent on Tak1 activation *in vitro*.

**Figure 8:**
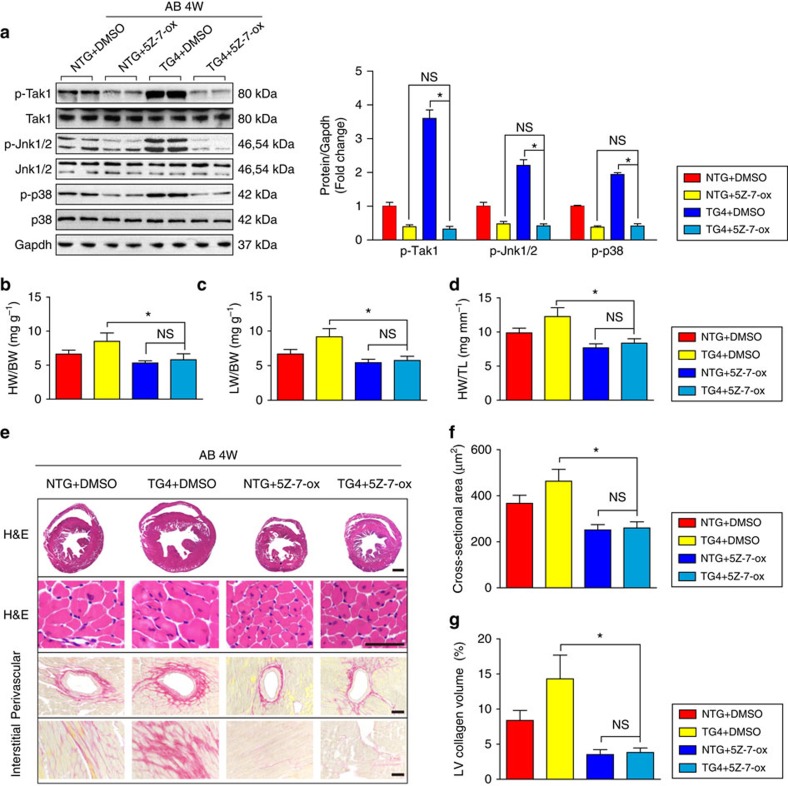
Inhibition of Tak1 abolishes the pro-hypertrophic effect of Traf6 overexpression *in vivo*. (**a**) Left, Western blots showing the phosphorylation of Tak1 and its downstream kinases in both NTG and *Traf6*-TG4 mice after AB surgery with or without 5z-7-ox treatment. Right, statistical results of the Tak1, Jnk1/2, and p38 phosphorylation levels in the indicated groups (*n*=4 mice per group). (**b**–**d**) The HW/BW (**b**) LW/BW (**c**) and HW/TL (**d**) ratios in the indicated groups following 4 weeks of AB treatment (*n*=12–13 mice per group). (**e**–**g**) Heart sections were stained with HE or PSR (**e**) to measure cardiomyocyte size (**f**) and fibrotic area (**g**) in the indicated groups (*n*=6–8 mice per group; scale bar, 1,000 μm for the upper most set of panels and scale bar, 50 μm for lower panels). In (**a**–**g**) *P<0.05 versus TG4 DMSO, NS, not significant. Data are presented as the mean±s.d. from at least three independent experiments. Statistical analysis was carried out by one-way ANOVA.

The Tak1-dependent effect of Traf6 on cardiac hypertrophy *in vitro* prompted us to evaluate whether the activation of Tak1 is responsible for Traf6-mediated cardiac hypertrophy *in vivo*. Thus, *Traf6*-TG and NTG mice were treated with 5z-7-ox (an inhibitor of Tak1) or DMSO (the same volume as the 5z-7-ox solution) as a control. As shown in Fig. 8a, 5z-7-ox successfully inhibited Tak1 phosphorylation after AB surgery in both TG and NTG mice. The blockage of Tak1 markedly attenuated the AB-induced cardiac hypertrophy (Fig. 8b–f), fibrosis (Fig. 8g) and cardiac dysfunction (Supplementary Fig. 6a) induced by pressure overload stimulation for 4 weeks in NTG mice. Notably, 5z-7-ox treatment largely abolished the enhanced hypertrophic response caused by Traf6 overexpression, leading to comparable cardiomyocyte size, fibrosis area and cardiac function between Tak1 inhibitor-treated TG and NTG mice (Fig. 8b–g; Supplementary Fig. 6a,b). Taken together, these data indicate that the activation of TAK1-JNK1/2/p38 signalling appears to be essential for TRAF6-mediated cardiac hypertrophy.

### TRAF6 auto-ubiquitination is required for TAK1 activation

**Figure 9:**
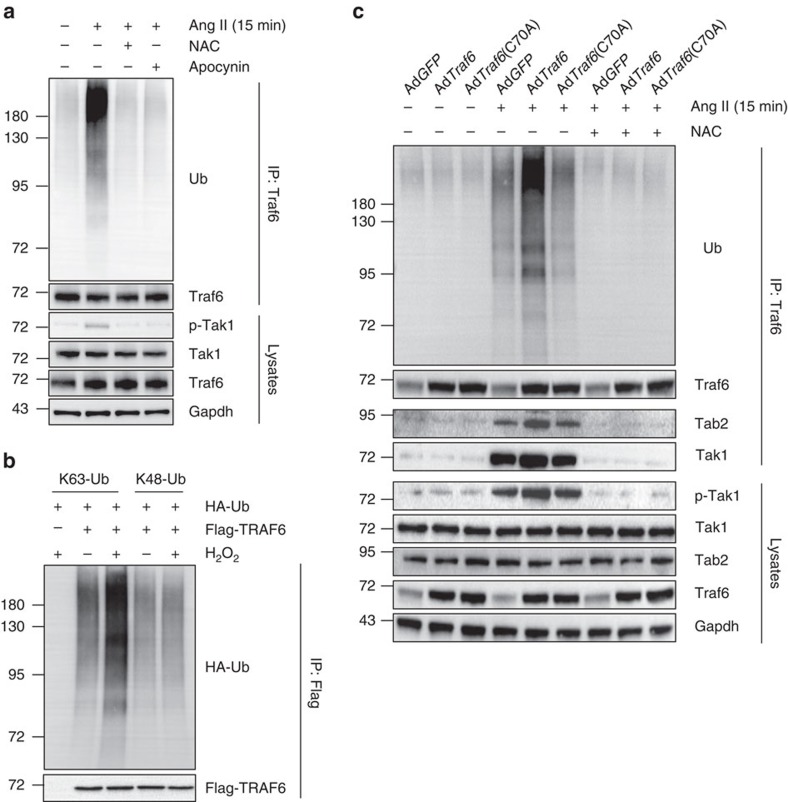
The ubiquitination of TRAF6 during cardiac hypertrophy is regulated by ROS production. (**a**) The ubiquitination of Traf6 examined by *in vivo* ubiquitination assay in NRCMs treated with Ang II for 15 min with or without NAC administration. The assay was performed with an anti-TRAF6 antibody followed by western blotting with an anti-Ub antibody. (**b**) HEK293T cells were transfected with HA-tagged K63-Ub or K48-Ub and FLAG-TRAF6, following H_2_O_2_ treatment for 15 min. (**c**) Primary cardiomyocytes were transfected with indicated adenovirus and treated with Ang II for 15 min in the presence or absence of NAC administration. *in vivo* ubiquitination assays and Co-IP experiments were performed using anti-TRAF6 antibody followed by anti-Ub (upper), anti-TAB2 or anti-TAK1 (middle) antibodies, respectively. At least three independent experiments were performed.

The requirement for TAK1 for the functional regulation of TRAF6 in cardiac remodelling brought about another important question: how does TRAF6 regulate the activation of TAK1? TAK1 activation can be attributed to various upstream molecular events^30^. Investigations into the activation of TRAF6 and its interaction with TAK1 might provide an answer to this question. Previous studies have indicated that the polyubiquitination of TRAF6 is closely related to TRAF6 activation^31^,^32^. Our study demonstrated that ROS production induced significant increase in Traf6 expression and activation (Figs 2, 3, 4, 5), but the involvement of TRAF6 ubiquitination in this process was unknown. Therefore, we investigated the level of Traf6 ubiquitination in NRCMs after Ang II stimulation. Figure 9a shows that Ang II treatment for 15 min significantly induced Traf6 ubiquitination and dramatically enhanced the level of phosphorylated Tak1. In the presence of NAC or APO, Traf6 ubiquitination and Tak1 phosphorylation were negligible (Fig. 9a). By transfecting HEK293T cells with HA-tagged K63 only-ubiquitin (K63-Ub) or K48-Ub, we further found that only K63-Ub but not K48-Ub allowed TRAF6 ubiquitination upon H_2_O_2_ stimulation (Fig. 9b). Additionally, the auto-ubiquitination of Traf6 disappeared after cardiomyocytes were infected with the Ad*Traf6* (C70A) mutant, in which the highly critical Cys residue in the RING domain was mutated to Ala and thus lost the E3 ligase activity (Fig. 9c). After ubiquitination, Traf6 recruits Tab2, a protein that can form a complex with Tak1 and participates in the activation of Tak1 (ref. 33). Consistently, our co-immunoprecipitation assay results revealed that ubiquitinated Traf6 can bind to Tab2 and Tak1, which was not observed with the Traf6 (C70A) mutant. Notably, binding of Traf6 to Tak1 was observed at the basal level, and this interaction was markedly enhanced by Ang II stimulation, along with the occurrence of Traf6 ubiquitination and the increased level of phosphorylated Tak1. The binding of Traf6 to Tab2 and the activation of Tak1 were markedly inhibited by NAC treatment (Fig. 9c). Thus, these studies indicated that after Ang II stimulation, the production of ROS induces Traf6 auto-ubiquitination and subsequent Tak1 activation.

### TRAF6–TAK1 interaction is essential for cardiac remodelling

**Figure 10:**
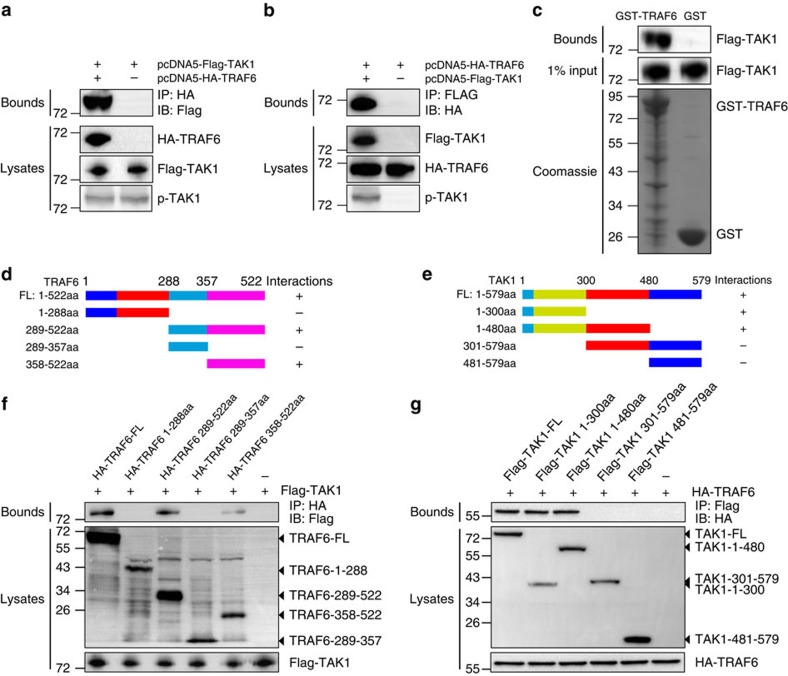
TRAF6 regulates TAK1 through direct physical interaction. (**a**,**b**) HEK293T cells were co-transfected with pcDNA5-FLAG-TAK1 and pcDNA5-HA-TRAF6. After 48 h, cells were harvested, and cellular lysates were subjected to IP with antibodies against HA (**a**) or FLAG (**b**). (**c**) GST–TRAF6 or GST was retained on glutathione–Sepharose beads, incubated with immunopurified Flag-TAK1 from HEK293T cells and then immunoblotted with the antibody against FLAG. (**d**,**e**) Schematic representation of the full-length and deletion mutants of TRAF6 (**d**) and TAK1 (**e**) used for determining the interaction domains. (**f**) Western blots performed using a FLAG or HA antibody after co-IP of the full-length or truncated forms of TRAF6 from HEK293T whole-cell lysates using the anti-HA antibody. (**g**) Representative western blot performed with a FLAG or HA antibody after co-IP of the full-length or truncated sequences of TAK1 from HEK293T whole-cell lysates with an anti-FLAG antibody.

After demonstrating the molecular events occurring with TRAF6 itself, we further sought to identify the intermediate mechanism linking TRAF6 ubiquitination and TAK1 activation. We first examined whether TRAF6 could physically interact with TAK1. To this end, we transfected FLAG-tagged TAK1 and HA-tagged TRAF6 into HEK293T cells and subjected cellular lysates to immunoprecipitation (IP). The ectopically expressed TRAF6 could interact with TAK1 and *vice versa*, but the TAK1 phosphorylation levels were indistinguishable between these two groups (Fig. 10a,b). Next, we performed a glutathione S-transferase (GST) pull-down assay with GST-tagged TRAF6 to determine whether the binding of TRAF6 to TAK1 occurs via a direct interaction. Consistent with the IP results, TAK1 was eluted along with TRAF6 (Fig. 10c). To explore the binding domains required for the TRAF6–TAK1 interaction, we constructed a series of TRAF6 and TAK1 deletion mutants (Fig. 10d,e) and observed that a region (aa 358 to 522) of TRAF6 and the amino-terminal 300 amino acids of TAK1 (N/aa 1–300) were required for the binding of TRAF6 to TAK1 (Fig. 10f,g).

**Figure 11:**
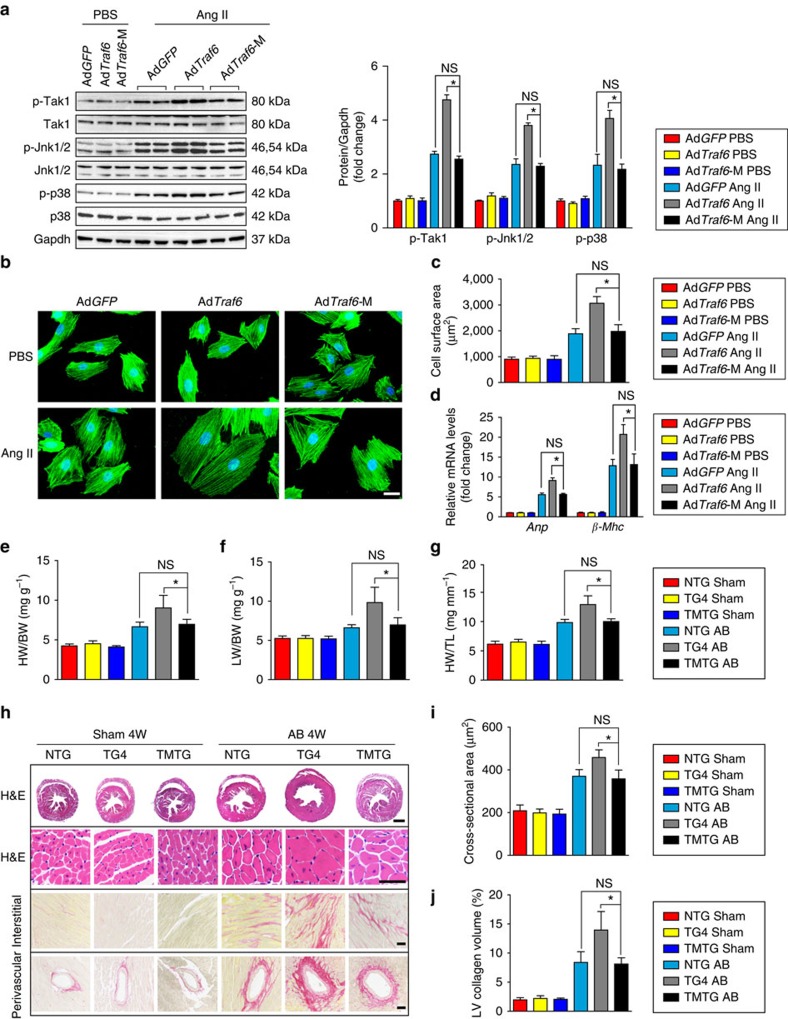
TRAF6-TAK1 interaction is required for TRAF6-mediated hypertrophic response. (**a**) Cardiomyocytes were induced to overexpress full-length Traf6 or a Tak1-binding-defective Traf6 and were then treated with PBS or Ang II (1 μM). The phosphorylation and total protein levels of Tak1, Jnk1/2, and p38 in the indicated groups. (**b**,**c**) Representative microscopic images (**b**) and cross-sectional areas (**c**, *n*>50 cells per group) of cardiomyocytes stained with the anti-α-actinin antibody after Ang II treatment for 48 h. Scale bars, 20 μm. (**d**) The mRNA expression of *Anp* and *β-Mhc* in the indicated groups (*n*=4 per group). For (**a**–**d**) *P<0.05 versus Ad*Traf6* Ang II, NS, not significant. (**e**–**g**) The HW/BW (**e**), LW/BW (**f**) and HW/TL (**g**) ratios were determined in the indicated groups 4 weeks after AB surgery (*n*=11–12 mice per group). (**h**–**j**) Representative images of heart slices stained with HE or PSR staining (**h**) and the calculated cross-sectional areas ((**i**) *n*>100 cells per group) and cardiac fibrosis (**j**, *n*>40 fields per group) at 4 weeks after AB surgery (*n*=6–7 mice per group; scale bar, 1,000 μm for the upper most set of panels and scale bar, 50 μm for lower panels). *P<0.05 versus TG4 AB, NS, not significant. Data are presented as the mean±s.d. from at least three independent experiments. Statistical analysis was carried out by one-way ANOVA.

The plasmid pcDNA3-Traf6-M, which encodes a Tak1-binding-defective Traf6 (referred to as ‘Traf6-M') lacking the Tak1-binding domain, was then generated to determine whether the interaction between Traf6 and Tak1 is a factor underlying the activation of Tak1 by Traf6 during the hypertrophic response. Traf6-M failed to activate Tak1-Jnk1/2 and p38 signalling (Fig. 11a); additionally, the capacity of Traf6 to regulate cardiomyocyte enlargement (Fig. 11b,c) and to increase mRNA levels of fetal genes (Fig. 11d) upon Ang II were lost, suggesting that the physical binding between Traf6 and Tak1 is essential for Tak1 activation and the resulting myocyte hypertrophy *in vitro*. To further determine whether the Traf6–Tak1 interaction is essential for the pro-hypertrophic effect of Traf6 *in vivo*, we generated a mouse strain with cardiac-specific overexpression of a Traf6 mutant with a Tak1-binding deficiency (hereafter referred to as TMTG; Supplementary Fig. 7a,b) and subjected NTG, TG and TMTG mice to AB surgery. Consistent with our observations *in vitro*, the TMTG mice did not significantly influence the development of cardiac remodelling and exhibited a global phenotype and cardiac performance comparable to the NTG mice in response to pressure overload (Fig. 11e–j; Supplementary Fig. 7c). Collectively, these results show that the TRAF6–TAK1 interaction is essential for the pro-hypertrophic effect of TRAF6 both *in vivo* and *in vitro*.

### E3 ligase activity of TRAF6 is vital for cardiac remodelling

**Figure 12:**
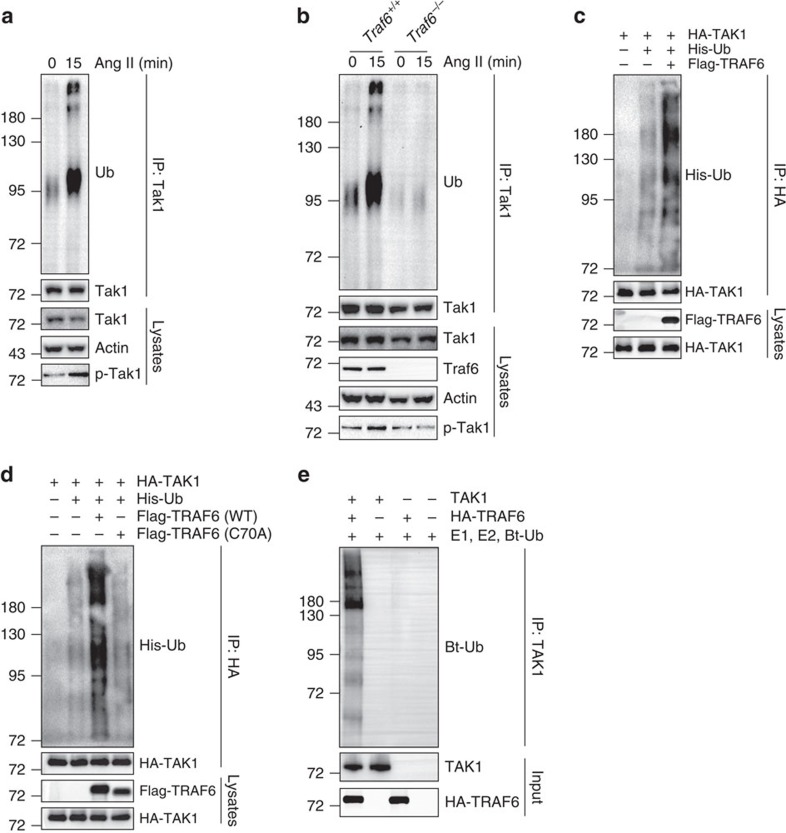
TAK1 is ubiquitinated by TRAF6. (**a**) NRCMs were stimulated with Ang II for 15 min and subjected to immunoprecipitation with anti-TAK1 antibody followed by western blotting with anti-Ub antibody. (**b**) Neonatal ventricular cells isolated from *Traf6*-CKO mice and their WT littermates stimulated with Ang II for 15 min. These experiments were performed as described in **a**. (**c**) HEK293T cells transfected with HA-TAK1 and His-Ub along with FLAG-TRAF6 plasmids were subjected to immunoprecipitation with anti-HA antibody followed by Western blotting with anti-Ub antibody. (**d**) HEK293T cells were transfected with HA-TAK1 and His-Ub along with TRAF6 (WT) or TRAF6 (C70A) plasmids. (**e**) TAK1 proteins were incubated with E1, E2 and biotinylated-Ub (Bt-Ub) in the presence or absence of TRAF6 proteins for *in vitro* ubiquitination of TAK1. Ubiquitination of TAK1 was analyzed by western blotting with streptavidin-HRP after immunoprecipitated using anti-TAK1 antibody.

Because TRAF6 possesses E3 ubiquitin ligase activity and the ubiquitination of TAK1 is imperative for its phosphorylation and subsequent activation^34^,^35^, we questioned whether TAK1 is ubiquitinated by TRAF6. To this end, we tested the ubiquitination of Tak1 in NRCMs upon Ang II stimulation and found that after 15 min of Ang II administration, the polyubiquitination of Tak1 was triggered (Fig. 12a). This polyubiquitination was markedly decreased when Traf6 was ablated (Fig. 12b), suggesting Traf6 is necessary for Tak1 ubiquitination. In HEK293T cells transfected with HA-TAK1 and His-Ub, introducing FLAG-TRAF6 induced significant TAK1 polyubiquitination (Fig. 12c). In contrast, the catalytically inactive TRAF6 (C70A) mutant, which has no E3 ligase activity, lost the ability to promote TAK1 ubiquitination (Fig. 12d). The catalytic ability of TRAF6 upon TAK1 ubiquitination was confirmed *in vitro* (Fig. 12e). In addition, by constructing a series of HA-tagged ubiquitin mutants, we found that TRAF6 could ubiquitinate TAK1 in the presence of K63 but not other ubiquitins, and the K63 mutation largely abolished TAK1 ubiquitination (Supplementary Fig. 8a). These results indicate that TRAF6 is an E3 ubiquitin ligase that mediates the K63-linked ubiquitination of TAK1 in response to hypertrophic stimuli.

**Figure 13:**
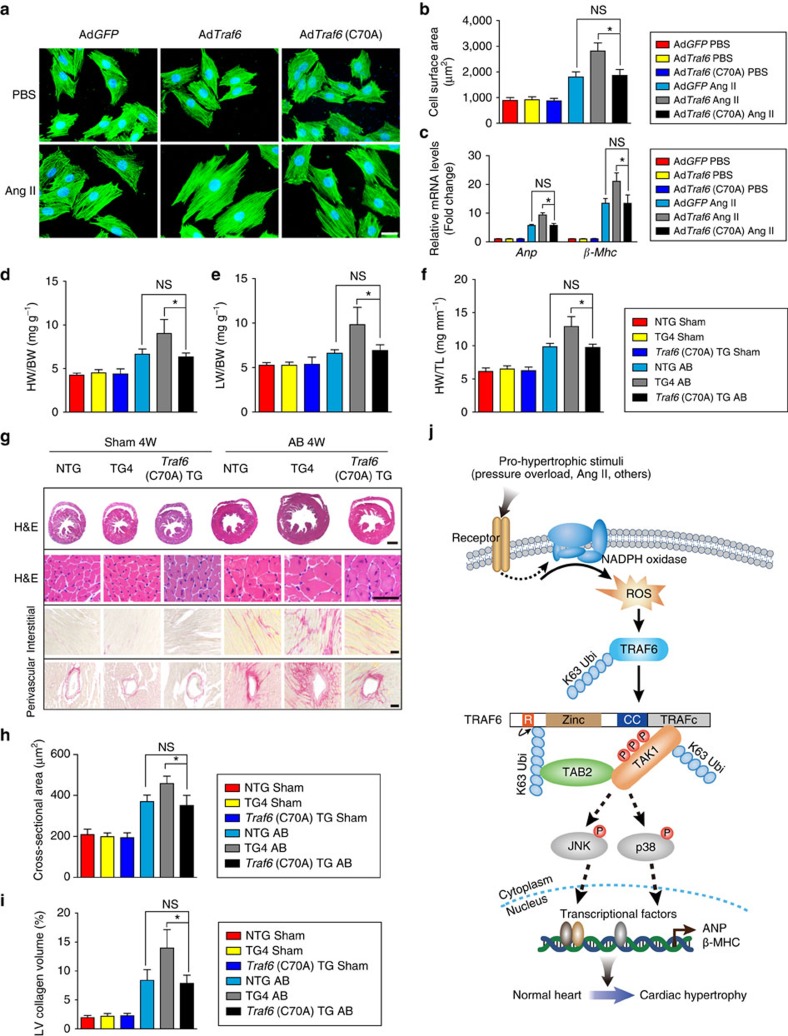
TRAF6-triggered TAK1 ubiquitination is essential for cardiac hypertrophy. (**a**,**b**) Representative images (**a**) and the cross-sectional areas ((**b**) *n*>50 cells per group) of cardiomyocytes stained with the α-actinin antibody. Indicated adenovirus-infected cells were stimulated with Ang II for 48 h (scale bar, 20 μm). (**c**) The mRNA levels of *Anp* and *β-Mhc* in the indicated adenovirus-infected cardiomyocytes after treatment with PBS or Ang II for 48 h. For (**b**,**c**) *P<0.05 versus Ad*Traf6* Ang II, NS, not significant. (**d**–**f**) The HW/BW (**d**) LW/BW (**e**) and HW/TL (**f**) ratios in the indicated groups following 4 weeks of AB treatment (*n*=10–13 mice per group). (**g**) Representative HE staining images of heart sections from mice in the indicated groups (*n*=6–8 mice per group; scale bar, 1,000 μm for the upper most set of panels and scale bar, 50 μm for lower panels). (**h**) Statistical results for the cross-sectional areas of cardiomyocytes in the indicated groups (*n*>100 cells per group). (**i**) The fibrotic areas in the indicated groups (*n*>40 fields per group). For (**d**–**h**) *P<0.05 versus TG4 AB, NS, not significant. Data are presented as the mean±s.d. from at least three independent experiments. Statistical analysis was carried out by one-way ANOVA. (**j**) Schematic diagram of the molecular mechanisms underlying TRAF6-regulated cardiac hypertrophy. R, RING domain; Zinc, zinc fingers; CC, coiled-coil domain; TRAFc, TRAF-C domain.

We next tested whether the catalytic activity of TRAF6 on TAK1 ubiquitination was essential for its pro-hypertrophic role by examining the effect of the Traf6 (C70A) mutation on hypertrophic models. As shown in Fig. 13a,b, Traf6 (C70A) overexpression had equivalent effects on Ang II-induced myocyte hypertrophy on NRCMs transfected with Ad*GFP*, suggesting that the catalytic activity was required for the pro-hypertrophic role of Traf6. To confirm this observation under physiological conditions *in vivo*, we generated mice with cardiac-specific overexpression of Traf6 (C70A). At baseline, Traf6 (C70A) TG mice did not show obvious cardiac abnormalities (Fig. 13d–i; Supplementary Fig. 8b). Four weeks after aortic banding, we observed that Traf6 (C70A) TG mice failed to exhibit the augmented hypertrophic growth and cardiac dysfunction that have been observed in *Traf6*-TG mice (Fig. 13d–i; Supplementary Fig. 8b), indicating that the E3 ligase activity of Traf6 is essential for Tak1 activation and the cardiac hypertrophic response.

## Discussion

The present study reveals the previously unrecognized biological function of TRAF6 in cardiac hypertrophy. During the development of pathological cardiac remodelling, a progressive increase in TRAF6 expression was induced by ROS production. Based on various *in vivo* and *in vitro* cardiac hypertrophy models, we clearly identified cardiac TRAF6 as a positive regulator of functional and structural cardiac remodelling via activating TAK1-JNK1/2/p38 signalling. Importantly, the increased TRAF6 expression and the activation of the TAK1-JNK1/2/p38 cascade were confirmed in failing human hearts. Mechanistically, in response to various pro-hypertrophic stimuli, the activation of NADPH oxidase and the production of ROS trigger TRAF6 auto-ubiquitination, which recruits TAB2 and promotes the formation of a TAK1 activation complex, directly enhancing the binding of TRAF6 to TAK1, leading to the TAK1 ubiquitination. The resulting TAK1 ubiquitination leads to increased phosphorylation of TAK1 and the activation of the downstream JNK and p38 cascades (Fig. 13j). The TRAF6–TAK1 interaction and the ubiquitination of TAK1 are required for TRAF6-regulated pathological cardiac hypertrophy.

TRAF6 functions as an important adaptor molecule in various signalling pathways and possesses a broad spectrum of substrates^11^,^12^,^13^. Ubiquitination is an important prerequisite for TRAF6 activation^36^. In our search for trigger(s) of TRAF6 activation during development of pathological cardiac hypertrophy, we focused on the production of ROS because of its key role in various cardiovascular diseases, including Ang II- or pressure overload-induced cardiac hypertrophy^20^,^21^,^37^,^38^. In the present study, we observed a significant increase in ROS content in hypertrophic hearts, along with dramatically increased NADPH oxidase activity, a major source of ROS in the cardiomyocytes. The generated ROS subsequently induced marked TRAF6 auto-ubiquitination and contributed substantially to its elevated function in the development of cardiac remodelling. Interestingly, the polyubiquitination of TRAF6 was not observed in its RING domain mutant, TRAF6 (C70A), indicating that an intact TRAF6 RING domain is required for its auto-ubiquitination and activation, as well as its resulting capacity to regulate downstream molecular signalling upon pro-hypertrophic stimuli. The potent effects of ROS production on the expression, ubiquitination and activation of TRAF6 demonstrated in our present study are consistent with previous reports that intracellular ROS can activate TRAF6 (refs 22, 23, 24).

The context- and/or cell type-specific biological functions of TRAF6 rely on the regulation of specific downstream factors^26^. The MAPK pathway is a well-documented signalling that is closely involved in the cardiac hypertrophic process and is downstream of TRAF6 (refs 11, 35, 39). In the current study, utilizing cardiac-specific genetic manipulation, we provided robust evidence suggesting that TRAF6 promotes the activation of TAK1, a MAPK kinase kinase (MAPKKK) that controls the downstream JNK1/2 and p38 MAPK cascades, during cardiac hypertrophy. In our study, inhibiting Tak1 activation using a specific Tak1 inhibitor (5z-7-ox) significantly attenuated pressure overload-induced cardiac hypertrophy and dysfunction in NTG mice. Of note, 5z-7-ox treatment largely abolished the hypertrophic response enhancement caused by Traf6 overexpression, and Tak1 knockdown reversed the Traf6-regulated cardiomyocyte enlargement in response to Ang II stimulation in primary NRCMs. Consistently, TAK1 activation exaggerates hypertrophic growth in response to pressure overload^40^,^41^,^42^, and the cardiac-specific overexpression of active TAK1 was sufficient to induce cardiac hypertrophy and dysfunction during development^43^, indicating that TAK1 is a pivotal nodal point for the development of cardiac hypertrophy. Taken together, the ‘rescue' experiments to reverse the elevated function of Traf6 by downregulating Tak1 suggest a requirement for Tak1 activation in the development of cardiac remodelling regulated by Traf6.

In hypertrophied myocardium, the identification of TAK1 as a TRAF6-interacting protein that is mediated through TAB2 recruitment after TRAF6 ubiquitination further provides a mechanistic bridge linking the elevation of TRAF6 expression and TAK1 hyperactivation. The disassociation of TRAF6 and TAK1 failed to induce the activation of TAK1 and its downstream effectors JNK1/2 and p38. Consistent with our observations, TRAF6–TAK1 binding is indispensable for TAK1 activation in response to proinflammatory stimuli, which is augmented by the adaptor protein TAB2 (ref. 44). In most cases, activation of TAK1 signalling requires E3 ubiquitin ligase activity, which promotes the synthesis of polyubiquitination chains and the subsequent auto-phosphorylation of TAK1 (refs 26, 45). Here, we further investigated the necessity of TRAF6′s E3 ligase activity in its effects on TAK1 activation in response to hypertrophic stimuli. These data suggest that the ubiquitination-related hyperactivation of TAK1 is necessary for TRAF6-regulated cardiac remodelling.

However, the notion of the exacerbated function of upregulated TAK1 on cardiac hypertrophy was challenged by a recent study demonstrating that mice with cardiac-specific ablation of Tak1 develop spontaneous cardiac remodelling and heart failure^46^. This discrepancy in the regulation of cardiac remodelling by TAK1 has not been fully understood. Notably, an evidence must be considered during further studies on the functional role of TAK1 in cardiac remodelling: TAK1 is an indispensable regulator of a wide variety of physiological events, especially in development and the immune response^47^,^48^,^49^, and the presence of TAK1 is critical for the differentiation of cardiomyocytes from cardiac precursor cells^50^. Mice with a Tak1 deficiency are embryonic lethal and develop dilated vessels as well as a markedly reduced ability to regulate immune responses and cell death^51^,^52^. Thus, the combined evidence from previous reports and our present study indicate that TAK1 represents a sensitive central molecular node in the heart that requires fine-tuning but not complete ablation or overexpression to maintain cardiac homoeostasis.

Finally, although we clearly demonstrated that ROS-triggered TRAF6 ubiquitination and the followed activation of TAK1-JNK1/2/p38 signalling are responsible for TRAF6-regulated cardiac hypertrophy, the gap of how post-translational modifications of TRAF6 lead to activation of the E3 ligase needs to be further filled. Additionally, Paul *et al*.^53^ have reported that in starvation-induced skeletal muscle atrophy, Traf6 ablation reduced the expression of key regulators of autophagy, a response also critically involved in the development of cardiac dysfunction^54^. Therefore, whether the potential role of TRAF6 in autophagic activity contributes to pathological cardiac hypertrophy remains to be further elucidated.

In summary, we provide the evidence that endogenous TRAF6 is an essential molecular switch for the development of pathological cardiac hypertrophy, which is dependent on TRAF6 ubiquitination, TRAF6–TAK1 interactions, and the TRAF6-induced ubiquitination of TAK1. Thus, targeting TRAF6 and/or its interaction with TAK1 may represent promising strategies for reversing pathological cardiac hypertrophy.

## Methods

### Reagents

Antibodies against the following proteins were purchased from Cell Signaling Technology: AKT (4,691, 1:1,000 dilution), p-AKT^Ser473^ (4,060, 1:1,000 dilution), MEK1/2 (9,122, 1:1,000 dilution), p-MEK1/2^Ser217/221^ (9,154, 1:1,000 dilution), ERK1/2 (4,695, 1:1,000 dilution), p-ERK1/2^Thr202/Tyr204^ (4,370, 1:1,000 dilution), JNK (9,252, 1:1,000 dilution), p-JNK^Thr183/Tyr185^ (4,668, 1:1,000 dilution), p38 (9,212, 1:1,000 dilution), p-p38^Thr180/Tyr182^ (4,511, 1:1,000 dilution), TAK1 (4,505, 1:1,000 dilution), p-TAK1^Ser412^ (9,339, 1:1,000 dilution), TBK1 (3,013, 1:1,000 dilution), p-TBK1^Ser172^ (5,483, 1:1,000 dilution), PI3-kinase p85 (4,257, 1:1,000 dilution), p-PI3-kinase p85^Tyr458^ (4,228, 1:1,000 dilution), p-FAK^Tyr925^ (3,284, 1:1,000 dilution), TAB2 (3,715, 1:1,000 dilution), Actin (4,970, 1:1,000 dilution), and ubiquitin (3,933, 1:1,000 dilution). Antibodies against TRAF6 (sc7,221, 1:200 dilution), ANP (sc20158, 1:200 dilution), β-MHC (sc53090, 1:200 dilution) and p-ASK1^Ser966^ (sc101634, 1:200 dilution) were obtained from Santa Cruz Biotechnology (Dallas, TX, USA). An antibody against ILK (ab76468, 1:500 dilution) was obtained from Abcam (Cambridge, UK). An antibody against p-ILK (S343) (AP3679a, 1:1,000 dilution) was obtained from Abgent (San Diego, CA, USA). An antibody against ASK1 (GTX107921, 1:500 dilution) was purchased from Gene Tex (Irvine, California, USA). Antibodies against GAPDH (MB001, 1:10,000 dilution) and FAK (BS3853, 1:500–1:1,000) were obtained from Bioworld Technology (Harrogate, UK). Antibodies against HA (H6908, 1:1,000 dilution) and FLAG (F3165, 1:1,000 dilution) were obtained from Sigma (St Louis, MO, USA). The BCA protein assay kit from Pierce (Rockford, IL, USA) was used for determination of protein concentrations. Peroxidase-conjugated secondary antibodies (Jackson ImmunoResearch Laboratories, at 1:10,000 dilution) were used for visualization of western blot bands. Fetal calf serum was purchased from HyClone (Waltham, MA, USA). Cell culture reagents and all other reagents utilized were purchased from Sigma (St Louis, MO, USA).

### Animal models and procedures

All the animal experimental protocols were approved by the Animal Care and Use Committee of Renmin Hospital of Wuhan University and were conducted in accordance with the National Institutes of Health (NIH) Guide for the Care and Use of Laboratory Animals.

Generation of tissue-specific *Traf6* knockout (*Traf6*-CKO) mice: *Traf6* flox mice (C57BL/6J background) were purchased from the European Mouse Mutant Archive (EMMA, project ID: EM08446). Mice carrying the *α-Mhc*-MerCreMer transgene (C57BL/6J background) were purchased from the Jackson Laboratory (stock no. 005650). *Traf6* flox mice were bred with Cre transgenic mice to generate *Traf6*^flox/flox^/MEM-Cre mice. For the cardiac-specific knockout of Traf6, tamoxifen (80 mg kg^−1^ per day, Sigma, T-5648) was injected into 6-week-old male *Traf6*^flox/flox^/MEM-Cre mice for 5 consecutive days.

Production of cardiac-specific Traf6, Traf6 (▵358–522) and Traf6 (C70A) transgenic mice: full-length *Traf6* cDNA, mutant *Traf6* (Δ358–522) or *Traf6* (C70A), respectively, was cloned downstream of the cardiac *α-Mhc* promoter. The linearized cardiac-specific plasmid was microinjected into fertilized mouse embryos to produce cardiac-specific mice that were identified by PCR analysis of tail genomic DNA. The PCR primers used were as follows: forward, 5′-ATCTCCCCCATAAGAGTTTGAGTC-3′ and reverse, 5′-GGGGACAATCCATAAGAGCA-3′. Male *Traf6*-CKO, *Traf6*-TG mice and their wild-type littermates, all aged 8–10 weeks (24–27 g), were used for all subsequent experiments.

Cardiac hypertrophy mouse models were established by AB-induced pressure overload^16^,^55^,^56^. Briefly, male mice were anaesthetised with pentobarbital sodium (50 mg kg^−1^, Sigma) by intraperitoneal injection. Adequate anaesthesia was confirmed by the absence of a toe pinch reflex. The left chest of each mouse was then opened at the second intercostal space to expose the thoracic aorta by blunting dissection. The exposed aorta was tied against a 27-Gauge (for body weights of 24–25 g) or 26-Gauge (for body weights of 26–27 g) needle with a 7–0 silk suture. After ligation, the needle was removed gently, and adequate aortic constriction was confirmed by Doppler analysis. Sham-operated mice underwent a similar procedure without ligation. All surgeries and subsequent analyses were performed in a blinded manner.

To examine the role of Traf6 in cardiac hypertrophy, we established a mouse model of cardiac hypertrophy induced by Ang II infusion. Ang II (1.4 mg kg^−1^ per day and dissolved in 0.9% NaCl) was subcutaneously infused for 4 weeks using an osmotic minipump (Alzet model 2004, Alza Corp) implanted into each mouse. Saline-infused animals served as infusion controls and were subjected to the same procedures as the experimental animals, with the exception of Ang II infusion.

To inhibit ROS production, male mice were treated with the ROS scavenger NAC (500 mg kg^−1^ per day) or APO (100 mg kg^−1^ per day) in the drinking water^37^,^57^. For Tak1 inhibitor treatment, the specific Tak1 inhibitor 5Z-7-oxozeaenol (5Z-7-ox; O9890–1 MG; Sigma, St Louis, MO, USA) was administered intraperitoneally to male non-transgenic (NTG) and *Traf6*-TG mice (5 mg kg^−1^) to inhibit Tak1 activation.

### Echocardiographic measurements

Echocardiography was performed to evaluate cardiac function at the indicated time points^2^,^58^,^59^. In brief, echocardiography was performed under continuous anaesthesia with 1.5–2% isoflurane using a Mylab30CV (ESAOTE) ultrasound system with a 15 MHz probe. M-mode tracings derived from the short axis of the LV at the level of the papillary muscles were recorded. The LVEDd and LVESd were measured at the largest and smallest LV areas, respectively, and the LVFS was calculated using the following formula: LVFS(%)=(LVEDd−LVESd)/LVEDd × 100%.

### Histological analysis

Four weeks after the induction of cardiac hypertrophy, the body weight of each mouse was recorded. Following euthanasia, the heart, lung and tibia were collected for further analysis. To assess myocyte hypertrophy and cardiac fibrosis, the hearts were fixed in 10% formalin and embedded in paraffin by standard histological protocols. Subsequently, these hearts were sectioned and stained with HE or PSR. The cross-sectional areas of myocytes and fibrotic areas were measured using a digital image analysis system (Image-Pro Plus, version 6.0) from images captured from HE- and PSR-stained sections.

### Cell culture and recombinant adenovirus generation

For the *in vitro* studies, NRCMs were isolated from 1-day-old Sprague-Dawley rats^60^,^61^. Briefly, cardiac cells were isolated in PBS containing 0.03% trypsin and 0.04% collagenase type II from the hearts of neonatal rats. Subsequently, NRCMs were purified by removing cardiac fibroblasts via a differential attachment technique. NRCMs were then seeded at a density of 2 × 10^5^ cells per well in six-well culture plates. After 48 h, the culture medium was replaced with serum-free DMEM/F12 for 12 h before stimulation with Ang II (1 μM) or PE (50 μM). For cardiomyocytes treated with the NADPH oxidase inhibitor or ROS scavenger, APO (100 μM), NAC (10 mM) or CAT (400 U ml^−1^) were added into the media before Ang II administration^62^,^63^.

We constructed adenoviruses carrying sequences encoding rat Traf6 (Ad*Traf6*), Tak1-binding-defective mutant Traf6 (Ad*Traf6*-M), E3 ligase activity catalytically inactive Traf6 mutant (Ad*Traf6* (C70A)), short hairpin RNA-targeting Traf6 (Adsh*Traf6*), constitutively active Tak1 (Adca*Tak1*) and dominant-negative Tak1 (Addn*Tak1*). Similar adenoviral vectors encoding the green fluorescent protein GFP gene (Ad*GFP*) and short hairpin RNA (AdshRNA) served as controls. NRCMs were infected with the corresponding adenoviruses at a multiplicity of infection (MOI) of 100 particles per cell for 24 h, followed by subsequent experiments.

### Immunofluorescence analysis

The cell surface area of NRCMs was assessed by immunofluorescent staining. Briefly, cardiomyocytes were treated with Ang II or PE for 48 h after infection with corresponding adenoviruses for 24 h. The cells were subsequently fixed with 3.7% formaldehyde, permeabilized with 0.1% Triton X-100 in PBS for 45 min and stained with α-actinin (05-384, Merck Millipore, 1:100 dilution), followed by a fluorescent secondary antibody. The surface areas were measured using Image-Pro Plus 6.0 software. The ROS contents in the heart were measured using DHE staining (5 μM; Invitrogen, Waltham, MA, USA) on fresh frozen heart sections (5 μm) according to the manufacturer's instructions. Images were captured using a special OLYMPUS DP72 fluorescence microscope (model BX51TRF).

### Immunohistochemical analysis

For immunohistochemistry, paraffin-embedded hearts were cut transversely into 5-μm sections. After a 5-min high-pressure antigen retrieval process in citrate buffer PH 6.0, the heart sections were blocked with 10% bovine serum albumin (BSA) for 60 min and subsequently incubated overnight at 4 °C with the primary antibodies. Binding was visualized with the appropriate peroxidase-conjugated secondary antibodies (A21020, Abbkine) for 60 min at 37 °C.

### Intracellular ROS measurement

Intracellular ROS production was examined using 2,7-dichlorodihydrofluorescein diacetate (DCF-DA, Invitrogen) as an indicator^64^,^65^. In brief, fresh heart tissues or NRCMs were homogenized in assay buffer followed by incubation with DCF-DA at 37 °C for 3 h. The fluorescence was quantified using a spectrofluorometer with an excitation wavelength of 488 nm and emission at 525 nm.

### NADPH oxidase activity

NADPH oxidase activity in heart tissue was assessed by lucigenin-enhanced chemiluminescence (50 μg of protein, 100 μM NADPH and 5 μM lucigenin)^64^,^66^. Diphenyleneiodonium was added to the reaction mixture as an inhibitor of NADPH oxidase. The relative light units of chemiluminescence were recorded over 10 min with a counting time of 5 s.

### Measurement of antioxidant enzyme activities

The activities of antioxidant enzymes, including GPx, CAT and SOD in the heart tissues of mice were assayed^67^,^68^.

GPx activity was examined by measuring the regeneration of GSH from GSSG based on the action of glutathione reductase with NADPH. The heart homogenates were added into 100 mM phosphate buffer containing 0.5 mM EDTA, 1.0 mM NaN_3_, 0.25 mM NADPH, 2.25 mM GSH and 1.0 U ml^−1^ glutathione reductase. The change of NADPH optical density was assayed through recording the absorbance at 340 nm for 2 min. GPx activity was calculated with a molar extinction coefficient for NADPH of 6.22 × 10^3^ M^−1^ cm^−1^.

The activity of CAT was measured through adding the tissue homogenates into 100 mM phosphate buffer containing 10 mM H_2_O_2_. Absorbance values were recorded at 240 nm for 30 s, and the CAT activity was expressed according to a H_2_O_2_ extinction coefficient of 43.6 × 10^3^ M^−1^ cm^−1^.

Total SOD activity was measured based on the reaction mixture including 0.1 mM EDTA, 10 mM acetylated cytochrome *c*, 50 mM hypoxanthine, and 8 mU xanthine oxidase in 50 mM potassium phosphate. Absorbance was read at 418 nm for 2 min. SOD activity was expressed as U per min per mg protein.

### Western blotting and quantitative real-time PCR

Total protein was extracted from heart tissues and primary cells in lysis buffer. Protein concentrations were determined using a Pierce BCA Protein Assay kit. Fifty micrograms of protein was subjected to SDS–polyacrylamide gel electrophoresis (PAGE; Invitrogen) and transferred to a polyvinylidene fluoride membrane (Millipore), followed by incubation with the corresponding primary antibodies overnight at 4 °C. After incubation with peroxidase-conjugated secondary antibodies (Jackson ImmunoResearch Laboratories, at 1:10,000 dilution), the bands were visualized using Bio-Rad ChemiDocTM XRS+ (Bio-Rad). Protein expression levels were normalized to corresponding GAPDH levels.

Total mRNA was extracted using TRIzol reagent (15596-026, Invitrogen) according to the manufacturer's instructions. mRNA was converted to cDNA using oligo (dT) primers with a Transcriptor First Strand cDNA Synthesis Kit (4897030001, Roche). Quantitative real-time PCR amplification of the indicated genes was performed using SYBR Green (O4887352001, Roche). Target gene expression was normalized to *Gapdh* gene expression. The primers for real-time PCR are shown in Supplementary Table 1.

### Plasmid constructs

GST-TRAF6 and pcDNA5-HA-TRAF6 were generated by cloning the human *TRAF6* gene into pGEX-4T-1 and pcDNA5-HA-C1, respectively. pcDNA5-HA-TAK1 and pcDNA5-FLAG-TAK1 were generated by cloning the human *TAK1* gene into pcDNA5-HA-C1 and pcDNA5-FLAG-C1, respectively. DNA fragments encoding various TRAF6 (1 to 288, 289 to 357, 289 to 522, and 358 to 522) and TAK1 (1 to 300, 1 to 480, 301 to 579, and 481 to 579) truncates were prepared by PCR and cloned into the psi-HA-C1 and psi-FLAG-C1 expression vectors, respectively. Wild-type *TRAF6* was PCR amplified from pcDNA5-HA-TRAF6 and cloned into psi-FLAG-C1. Site-directed mutagenesis was performed by PCR. The wild-type pCI-His-Ubi plasmid was obtained from Addgene (plasmid 31815). The wild-type HA-Ub and its mutants were gifts from Dr Hongbing Shu (Wuhan University, Wuhan, China). The primers for these constructs are shown in Supplementary Table 2. All plasmids were verified by sequencing.

### Immunoprecipitation and GST pull-down assays

Immunoprecipitation and GST pull-down assays were performed to determine protein–protein interactions^61^,^69^. For immunoprecipitation, cells were washed with cold PBS (Hyclone) and lysed with lysis buffer (20 mM Tris–HCl, pH 7.4, 150 mM NaCl, 1 mM EDTA, 1% Triton X-100) containing Protease Inhibitor Cocktail Tablets (04693132001, Roche). After being pre-cleared with normal mouse or rabbit immunoglobulin G and protein A/G-agarose beads (11719394001 and 11719386001, Roche), lysates were incubated with the indicated primary antibodies and Protein G-agarose (11243233001, Roche) at 4 °C overnight with gentle shaking. The immunoprecipitated proteins were further washed five times with lysis buffer, boiled with 2 × SDS loading buffer, separated with SDS–PAGE and electrophoretically transferred to polyvinylidene difluoride membranes (Millipore). The membranes were blocked with 5% BSA in Tris-buffered saline containing 0.1% Tween-20 and were immunoreacted with the indicated primary antibodies and secondary antibodies conjugated to HRP.

For the GST pull-down assay, immunopurified Flag-TAK1 was prepared according the IP assay followed by incubation in elution buffer (20 mM Tris–HCl, pH 7.4, 150 mM NaCl, 1 mM EDTA, 1% Triton X-100, 3 μg μl^−1^ FLAG peptide (F4799, Sigma)) for 2 h at 4 °C. The Rosetta (DE3) *Escherichia coli* was transformed with vectors pGEX-4T-1-GST-TRAF6, and then induced with 0.5 mM isopropyl-β-D-thiogalactopyranoside (IPTG) at an optical density at 600 nm (OD600) of 0.8. *E. coli* extracts were prepared in PBS containing protease inhibitor cocktail tablets (04693132001, Roche) and incubated with glutathione-Sepharose 4B beads (17075601, GE Healthcare Biosciences AB) for 1 h at 4 °C. Subsequently, the beads loaded with the proteins were washed five times with 1 ml of PBS and incubated for an additional 4 h at 4 °C with immunopurified Flag-TAK1. The glutathione-Sepharose 4B beads then were washed three times with 1 ml of the IP lysis buffer in the absence of cocktail, and the eluted proteins, in buffer containing 20 mM reduced glutathione were resolved via SDS–PAGE and analysed via western blotting using anti-FLAG antibodies. A GST tag was used as the negative control under the same conditions.

### *In vivo* ubiquitination assay

The *in vivo* ubiquitination assay was performed following the protocol described in previous studies^70^. Briefly, cells were lysed in SDS lysis buffer (20 mM Tris–HCl, pH 7.4, 150 mM NaCl, 1 mM EDTA, 1% SDS) containing Protease Inhibitor Cocktail Tablets (04693132001, Roche) and denatured by heating for 5 min. The supernatants were diluted 10-fold with lysis buffer (20 mM Tris–HCl, pH 7.4, 150 mM NaCl, 1 mM EDTA, 1% Triton X-100) containing Protease Inhibitor Cocktail Tablets (04693132001, Roche). After centrifugation at 20,000 r.p.m. for 30 min at 4 °C, the supernatants were subjected to immunoprecipitation with the indicated antibodies.

### *In vitro* ubiquitination assay

The tested proteins were expressed with a TNT Quick Coupled Transcription/Translation System Kit (L2080, Promega) following the manufacturer's instructions. For the *in vitro* ubiquitination assay, 5 nM TRAF6 and 1 μM TAK1 protein were mixed with 100 nM His-E1, 1 μM His-E2 (Ubc13/Mms2) and 2.5 μM Bt-Ub in 50 μM ubiquitination reaction buffer from the ubiquitination kit (BML-UW9920-0001, Enzo Life Sciences) according to the manufacturer's instructions. Samples were subsequently immunoprecipitated with an anti-TAK1 antibody (5,206, Cell Signaling Technology) and separated on SDS–PAGE followed by streptavidin conjugated to HRP (ab7403, Abcam).

### Human heart samples

Samples from failing human hearts were collected from the left ventricles of DCM patients and HCM patients. Control human heart samples were obtained from the left ventricles of healthy donors who died in accidents, and these hearts were not suitable for transplantation due to non-cardiac reasons. Written informed consent was obtained from the patients and families of the donors. All the procedures involving human samples were approved by the Renmin Hospital of Wuhan University Review Board in Wuhan, China, and conformed to the principles outlined in the Declaration of Helsinki. Detailed information of human heart samples are shown in Supplementary Table 3.

### Statistical analysis

Data are presented as the mean±s.d. from at least three independent experiments. Student's two-tailed *t*-test was used to compare the means of two-group samples, and a one-way analysis of variance (ANOVA) was applied for comparison of multiple groups, followed by the least significant difference (equal variances assumed) or Tamhane's T2 (equal variances not assumed) tests. All statistical analyses were performed with SPSS (Statistical Package for the Social Sciences) 13.0. software. *P* values less than 0.05 were considered significant. No statistical method was used to predetermine sample size. No data were excluded. Randomization and blinding strategy was used whenever possible. Animal cohort sizes were determined on the basis of similar previous studies.

## Additional information

**How to cite this article:** Ji, Y.-X. *et al*. The ubiquitin E3 ligase TRAF6 exacerbates pathological cardiac hypertrophy via TAK1-dependent signalling. *Nat. Commun.* 7:11267 doi: 10.1038/ncomms11267 (2016).

## Supplementary Material

Supplementary Information Supplementary Figures 1-9 and Supplementary Tables 1-3
