# Supplementary material for: The ubiquitin E3 ligase TRAF6 exacerbates pathological cardiac hypertrophy via TAK1-dependent signalling
Source: Nat Commun. 2016 Jun 1;7:11267. doi: 10.1038/ncomms11267 (PMC4895385; doi:10.1038/ncomms11267)
Supplement: Supplementary Information — Supplementary Figures 1-9 and Supplementary Tables 1-3 [file ncomms11267-s1.pdf]

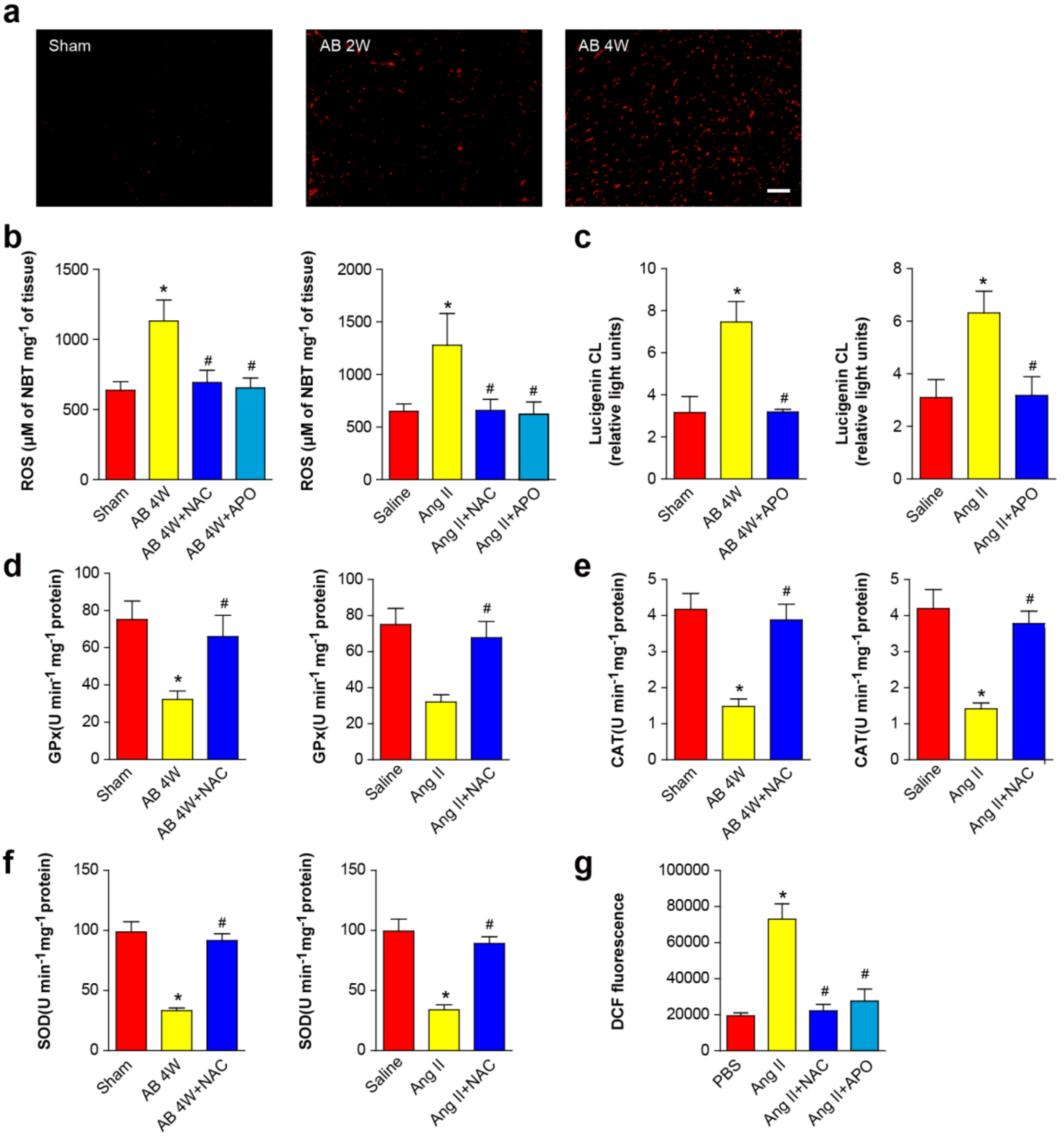

Supplementary Figure. 1. ROS production is increased during cardiac hypertrophy. (a) Representative image of DHE staining on the fresh frozen heart sections of WT mice after AB surgery for 2 or 4 weeks. (n=4 mice/group. Scale bar, 20 μm). **(b)** The contents of ROS in the heart samples from mice in the sham or AB surgery (**left panel**) or AngII infusion (**right panel**) groups with or without the ROS scavenger (N-acetyl-cysteine, NAC) or NADPH oxidase inhibitor (apocynin, APO) administration. **(c)** The activities of NADPH oxidase in the indicated groups after 4 weeks of pressure overload (**left**) or AngII infusion (**right**). **(d-f)** The activities of antioxidant enzymes glutathione peroxidase (GPx; **d**), Catalase (CAT; **e**), and superoxide dismutase (SOD; **f**) in the indicated groups after AB surgery or AngII infusion for 4 weeks. In **b-f**, n=12-18 mice/group; *P<0.05 vs. sham or Saline group, #P<0.05 vs. AB 4W or Ang II group. **(g)** The contents of intracellular ROS in NRCMs treated with Ang II in the presence or absence of NAC or APO. *P<0.05 vs. PBS, #P<0.05 vs. Ang II group. Data are presented as the mean±s.d. from at least three independent experiments. Statistical analysis was carried out by one-way ANOVA.

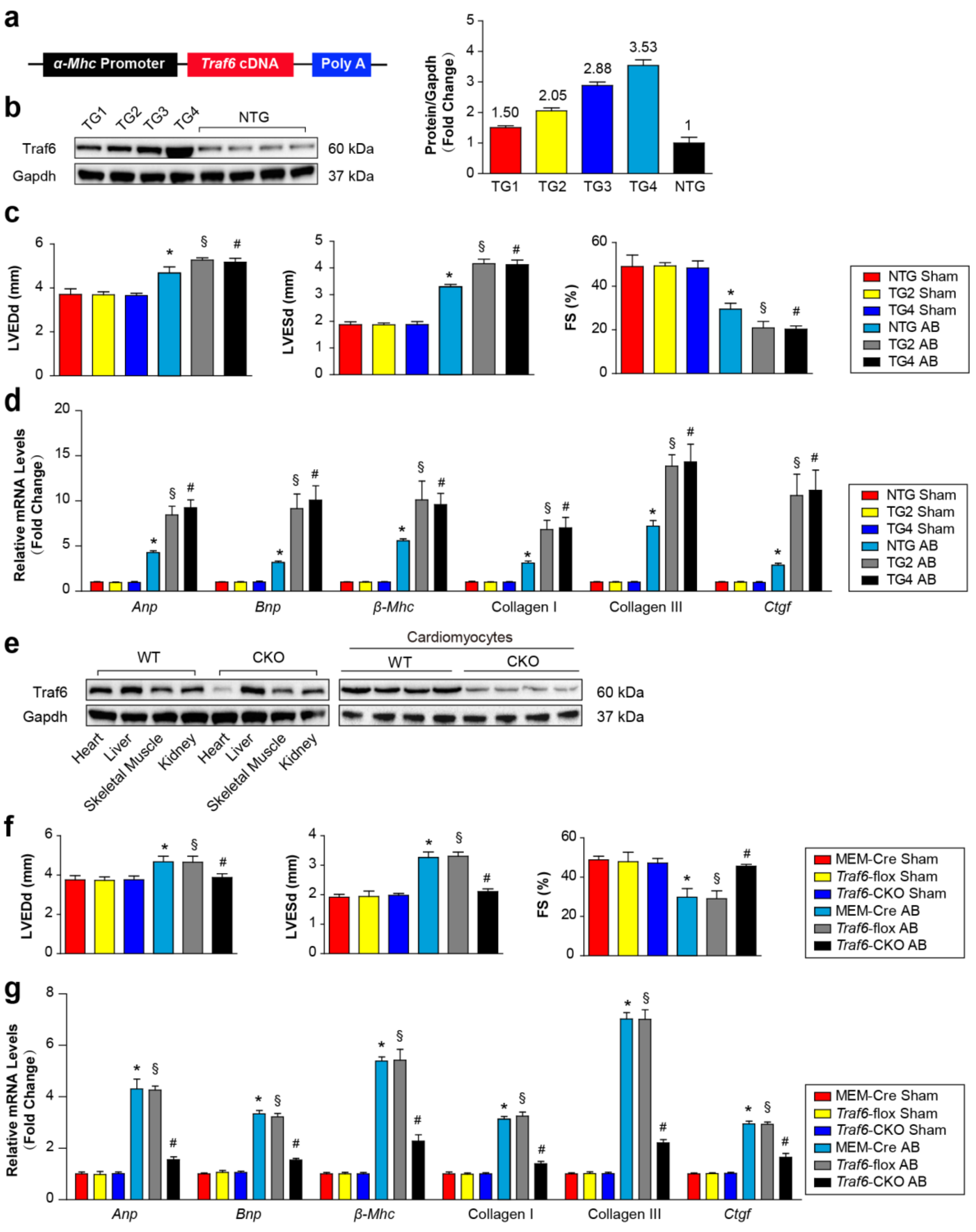

Supplementary Figure. 2. Traf6 promotes AB-induced cardiac hypertrophy. (a) Schematic diagram illustrating the construct used to generate *Traf6*-transgenic (TG) lines. **(b)** Overexpression of cardiac Traf6 was confirmed by Western blot analysis in TG mice compared with their non-TG (NTG) controls. n=3 independent experiments. **(c)** Echocardiographic measurements of left ventricle end-diastolic dimension (LVEDd), LV end-systolic dimension (LVESd) and LV fractional shortening

(FS) in different groups (n=6-7 mice/group). **(d)** The transcription levels of fetal genes and fibrotic markers in the heart tissues of NTG and TG mice after sham or AB surgery (n=6 mice/group. **P*<0.05 vs. NTG sham; §<0.05 vs. NTG AB, #*P*<0.05 vs. NTG AB). **(e)** Traf6 expression was determined in different tissues (**left panel**) or primary cardiomyocytes (**right panel**) of *Traf6*-CKO mice and their wild type (WT) controls. n=3 independent experiments. **(f)** Echocardiographic measurements of LVEDd, LVESd, and FS in different groups (n=6-7 mice/group). **(g)** The mRNA levels of fetal genes and fibrotic markers in the heart tissues of *Traf6*-CKO mice and WT controls after sham or AB surgery were determined by real-time quantitative PCR (n=4 mice/group). **P*<0.05 vs. MEM-Cre sham; §*P*<0.05 vs. *Traf6*-flox sham, #*P*<0.05 vs. *Traf6*-flox AB. Data are presented as the mean±s.d. from at least three independent experiments. Statistical analysis was carried out by one-way ANOVA.

.

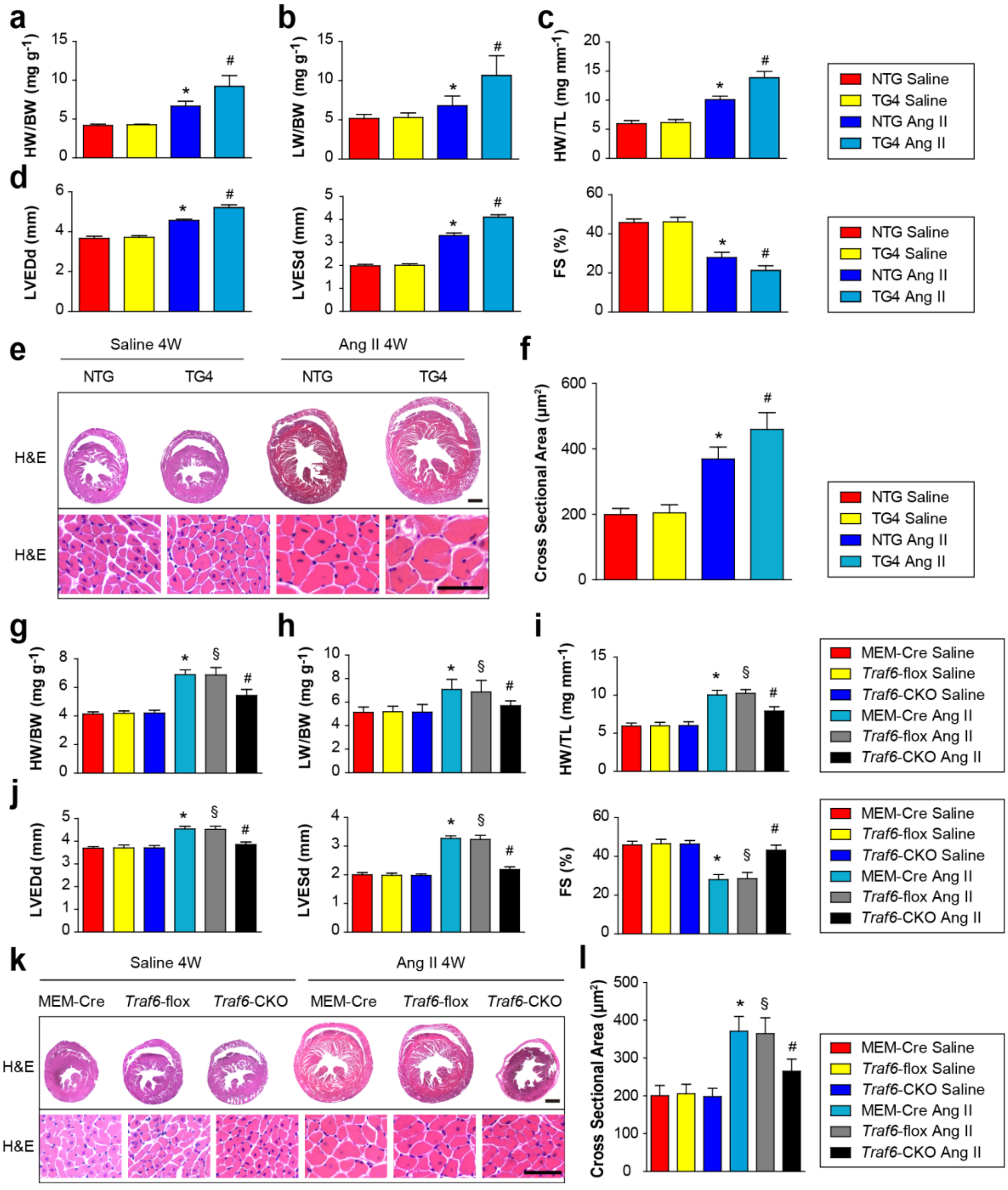

Supplementary Figure. 3. Traf6 promotes cardiac hypertrophy in mice infused with Ang II. **(a-c)** The HW/BW **(a)**, LW/BW **(b)**, and HW/TL **(c)** ratios were determined in the indicated groups 4 weeks after Ang II infusion (n=12-13 mice/group). **(d)** Echocardiographic measurements of LVEDd, LVESd, and FS in different groups (n=12-13 mice/group). **(e)** Histological analysis of heart slices by HE staining for the assessment of cardiomyocyte cross-sectional area 4 weeks after Ang II infusion (n=6-8 mice/group; scale bar, 1000 μm for the upper panels and scale bar, 50 μm for lower panels). **(f)** Statistical results for the cell cross-sectional areas in the indicated groups (n>100 cells/group). **(g-i)**

The HW/BW **(g)**, LW/BW **(h)**, and HW/TL **(i)** ratios were determined in *Traf6*-CKO and their littermate controls (MEM-Cre and *Traf6*-flox) 4 weeks after Ang II infusion (n=10-13 mice/group). **(j)** Echocardiographic measurements of LVEDd, LVESd, and FS in different groups (n=10-13/group). **(k)** Histological analysis of heart slices by HE staining to assess cardiomyocyte cross-sectional areas 4 weeks after Ang II infusion (n=6-8 mice/group; scale bar, 1000 μm for the upper panels and scale bar, 50 μm for lower panels). **(l)** Statistical results for the cell cross-sectional areas (n>100 cells/group). **P*<0.05 vs. MEM-Cre or NTG sham; §*P*<0.05 vs. *Traf6*-flox sham, #*P*<0.05 vs. *Traf6*-flox or NTG AB. Data are presented as the mean±s.d. from at least three independent experiments. Statistical analysis was carried out by one-way ANOVA.

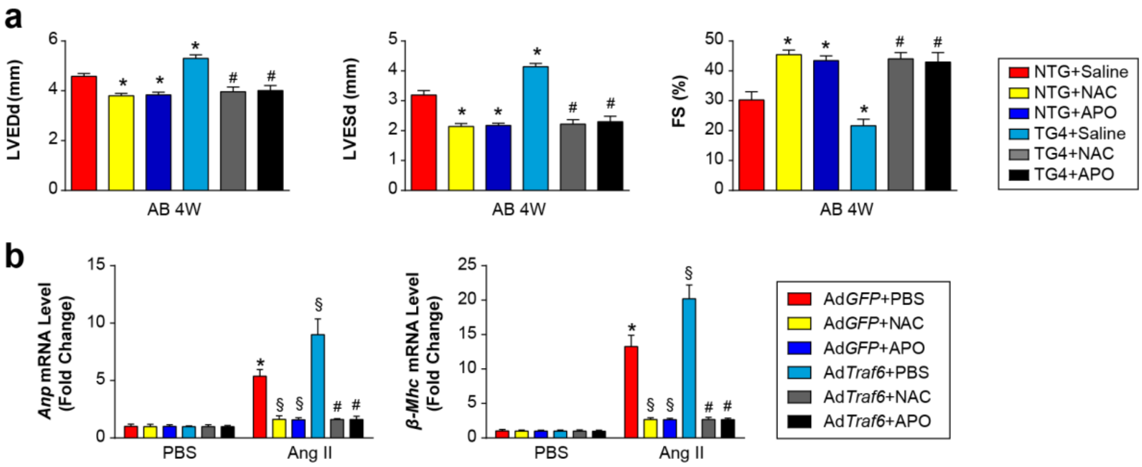

Supplementary Figure. 4. Blocking ROS reverses Traf6-regulated exacerbation of cardiac remodeling. (a) The values of LVEDd, LVESd, and FS of mice in the indicated groups at 4 weeks after pressure overload. (n=11-13 mice/group *P< 0.05 vs. NTG+saline group; #P<0.05 compared to TG4+saline group). **(b)** The mRNA levels of *Anp* and *β-Mhc* in NRCMs infected with Ad*GFP* or Ad*Traf6* and treated with PBS or Ang II in the presence or absence of NAC or APO. *P< 0.05 vs. Ad*GFP*/PBS+PBS group; §P<0.05 vs. Ad*GFP*/Ang II+PBS group; #P< 0.05 vs. Ad*Traf6*/Ang II+PBS group. Data are presented as the mean±s.d. from at least three independent experiments. Statistical analysis was carried out by one-way ANOVA.

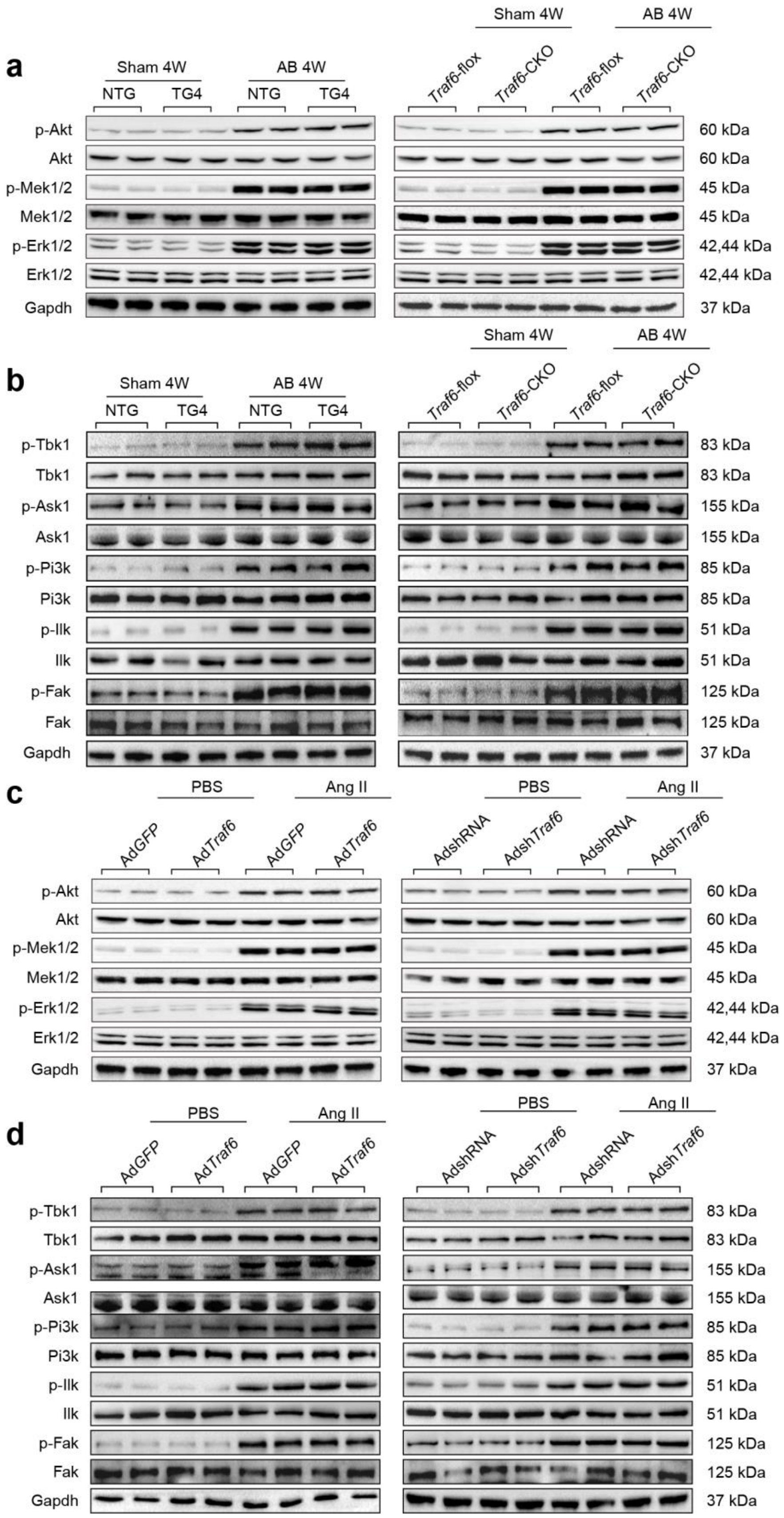

Supplementary Figure. 5. Traf6 mediates cardiac hypertrophy dependent on Tak1-Jnk1/2/p38

signaling. (a) Western blots showing the phosphorylation and total protein levels of Akt, Mek1/2, and Erk1/2 in heart tissues from NTG and TG4 mice **(left)** or *Traf6*-flox and *Traf6*-CKO mice **(right)** 4 weeks after AB surgery. **(b)** The phosphorylation and total protein levels of Tbk1, Ask1, Pi3k, Ilk, Fak in heart tissues from NTG and TG4 mice **(left)** or *Traf6*-flox and *Traf6*-CKO mice **(right)** mice subjected to sham or AB surgery. For **a** and **b**, n=4 mice/group. **(c, d)** The phosphorylation and total protein levels of Akt, Mek1/2, Erk1/2, and potential upstream factors of Jnk/p38 cascades in Ang II-treated NRCMs infected with Ad*GFP* and Ad*Traf6* **(left)** or AdshRNA and Adsh*Traf6* **(right)**. All data are representative of at least three independent experiments.

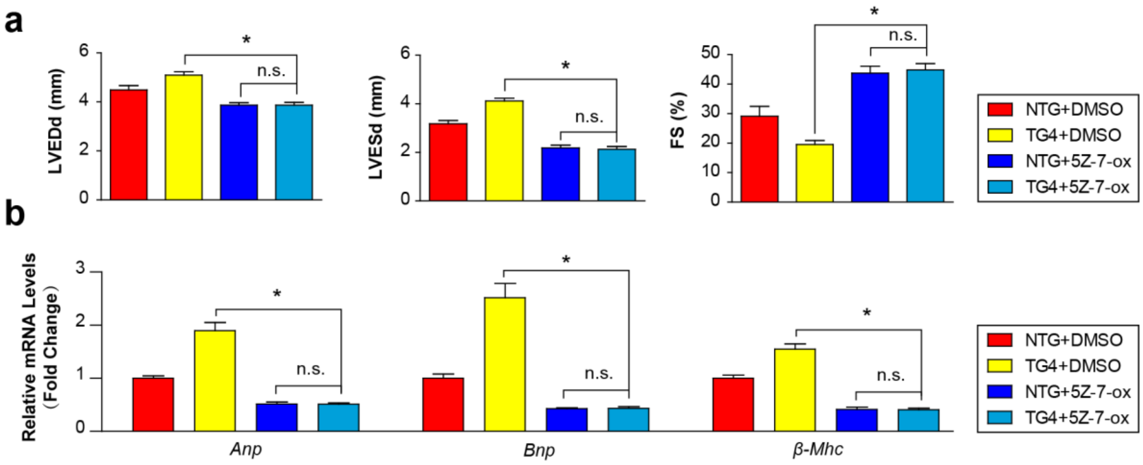

Supplementary Figure. 6. Inhibition of Tak1 abolishes the pro-hypertrophic effect of Traf6 overexpression *in vivo*. (**a**) Echocardiographic measurements of LVEDd, LVESd, and FS in different groups (n=6-7 mice/group). **(b)** The transcription levels of the fetal genes *Anp*, *Bnp*, and *β-Mhc* in the heart tissues of the indicated groups after AB surgery (n=4 mice/group). *$P<0.05$ vs. TG4 DMSO, n.s. not significance. Data are presented as the mean±s.d. from at least three independent experiments. Statistical analysis was carried out by one-way ANOVA.

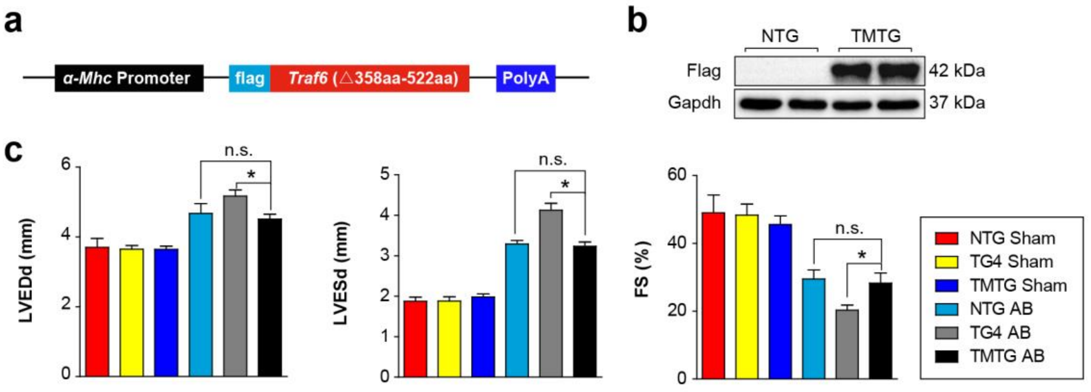

Supplementary Figure. 7. The Traf6-Tak1 interaction is required for Traf6-mediated hypertrophic response *in vitro*. (a) Schematic diagram illustrating the construct used to generate *Traf6* (△358aa-522aa)-transgenic (TG) mice. **(b)** Expression of cardiac mutant Traf6 was confirmed by Western blot analysis. n=3 independent experiments. **(c)** Echocardiographic measurements of LVEDd, LVESd, and FS in the indicated groups (n=6-10 mice/group). *$P<0.05$ vs. TG4 AB, n.s. not significance. Data are presented as the mean ± s.d. from at least three independent experiments. Statistical analysis was carried out by one-way ANOVA.

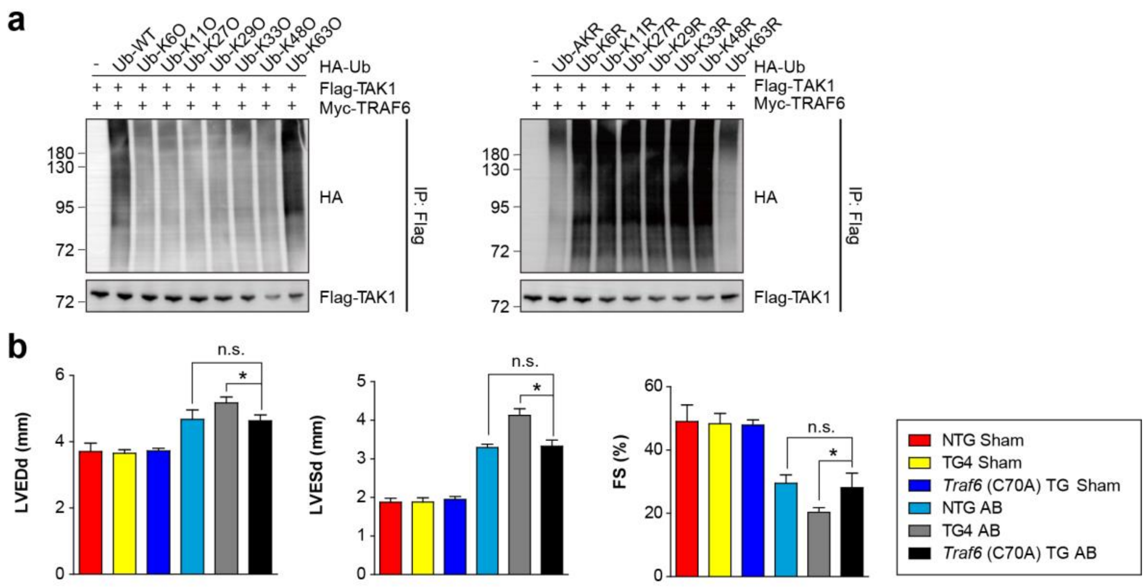

Supplementary Figure. 8. The E3 ligase activity of TRAF6 is essential for TAK1 activation and cardiac hypertrophic. (a) The ubiquitination of TAK1 measured in HEK293T cells infected with indicated HA-Ub mutant with Flag-TAK1 and Myc-TRAF6. Ub-WT, Ub-wildtype; Ub-K6O, Ub-lysine(6) only; Ub-AKR, Ub-All lysine to arginine; Ub-K6R, Ub-lysine(6) to arginine. **(b)** Echocardiographic measurements of LVEDd, LVESd and FS in different groups (n=6-10 mice/group). *P<0.05 vs. TG4 AB, n.s. not significance. Data are presented as the mean±s.d. from at least three independent experiments. Statistical analysis was carried out by one-way ANOVA.

Fig.1a

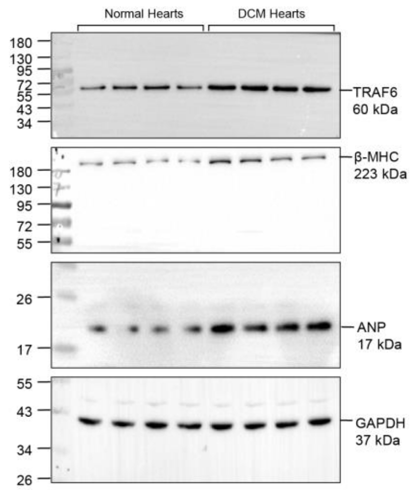

Fig.1b

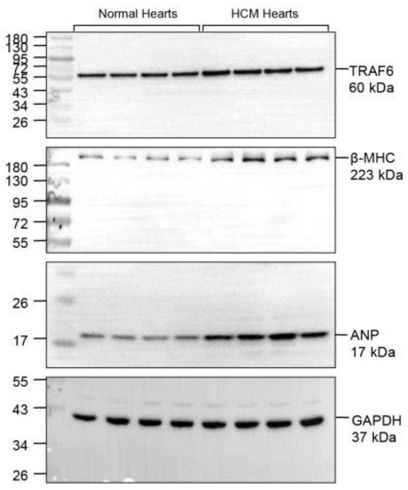

Fig.1c

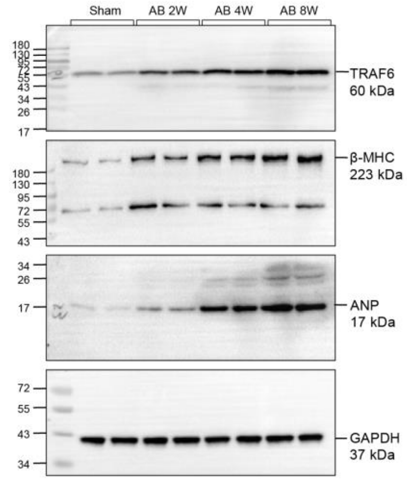

Fig.1d

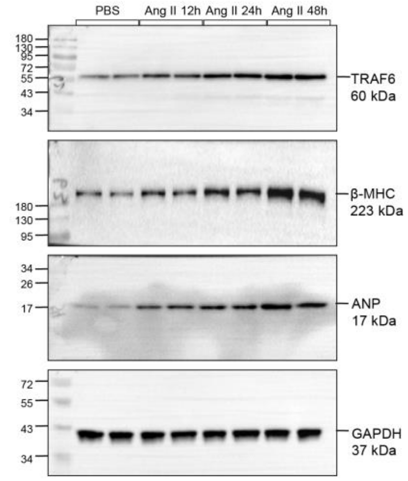

Fig.1e

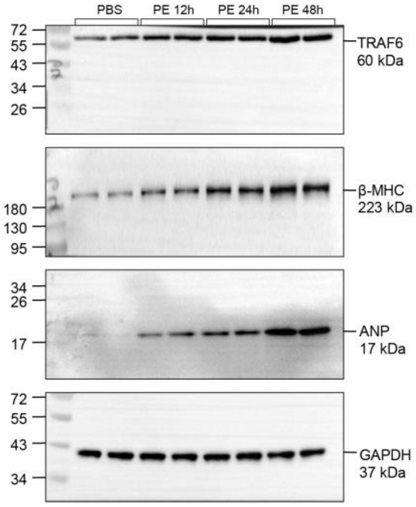

Supplementary Figure. 9. Full gel scans relating to indicated figures.

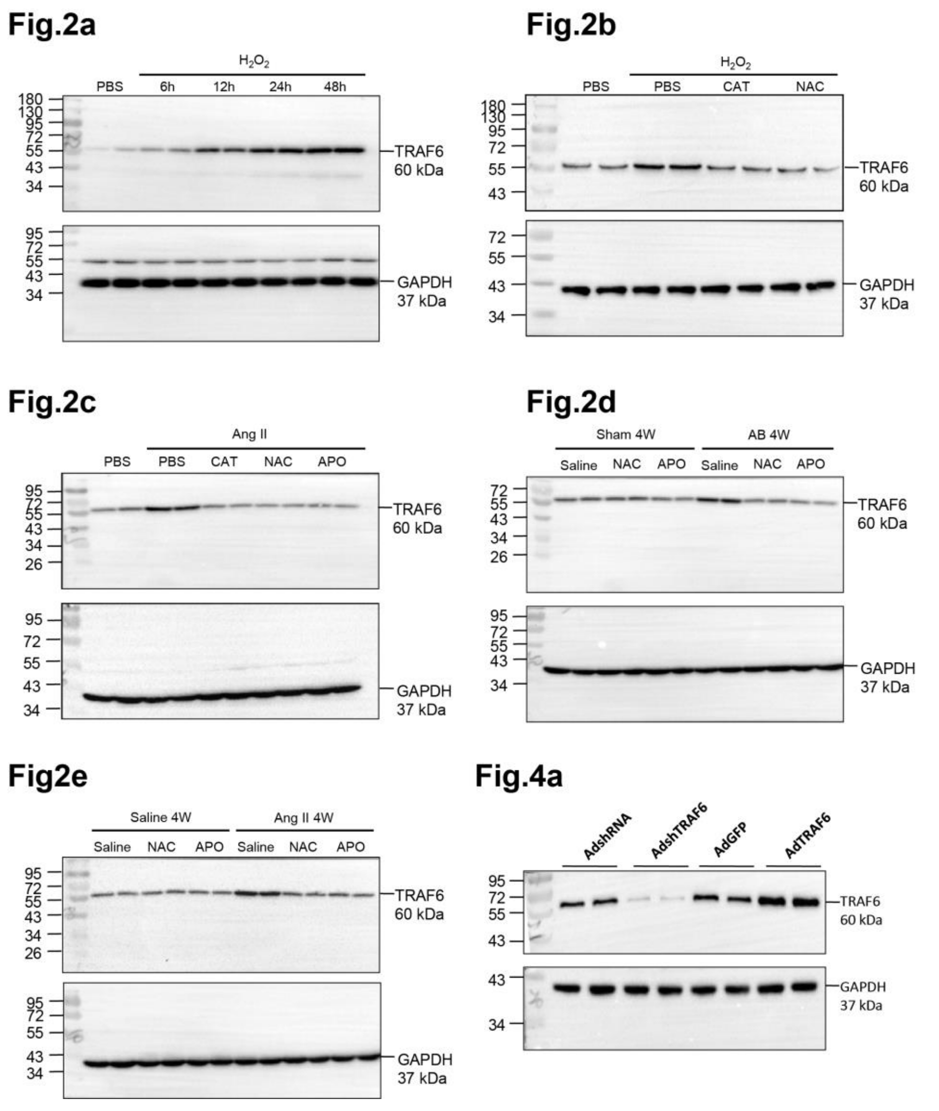

Supplementary Figure. 9. Full gel scans relating to indicated figures (continued).

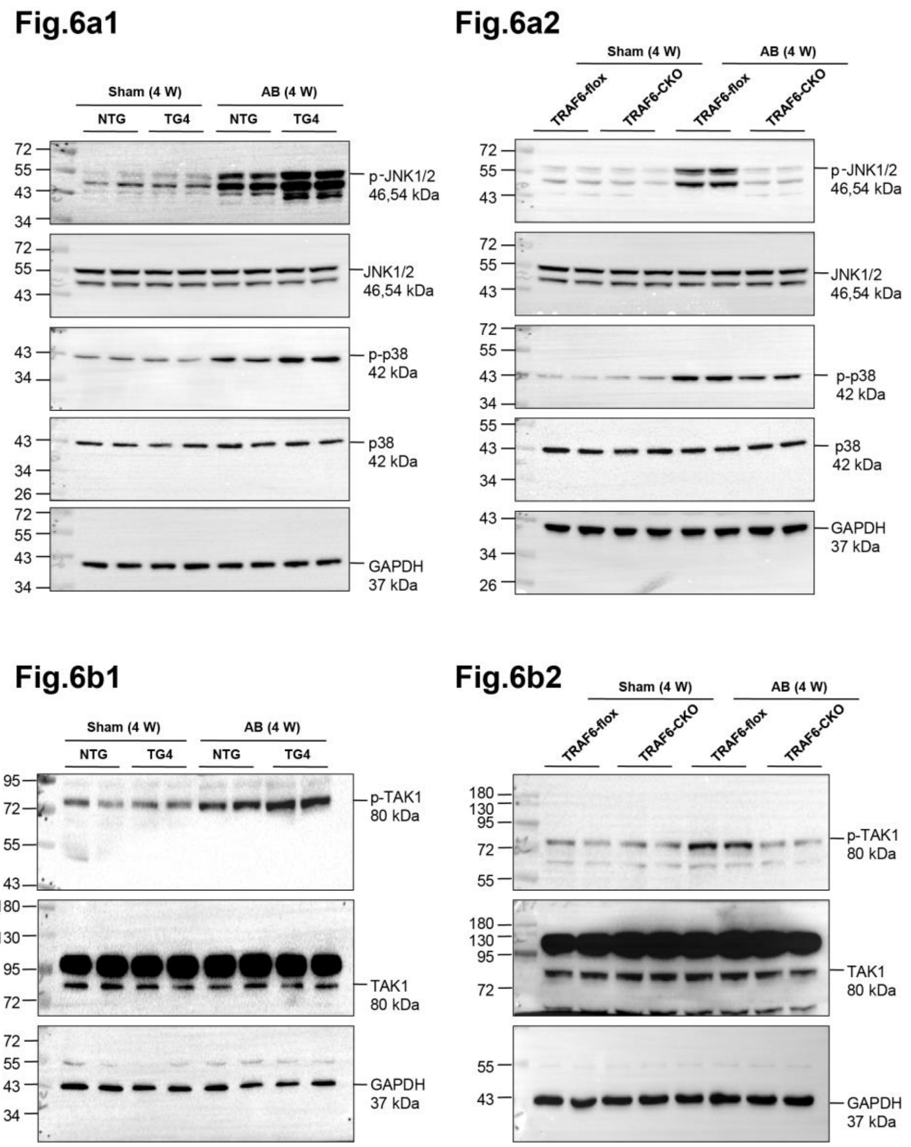

Supplementary Figure. 9. Full gel scans relating to indicated figures (continued).

Fig.6c1

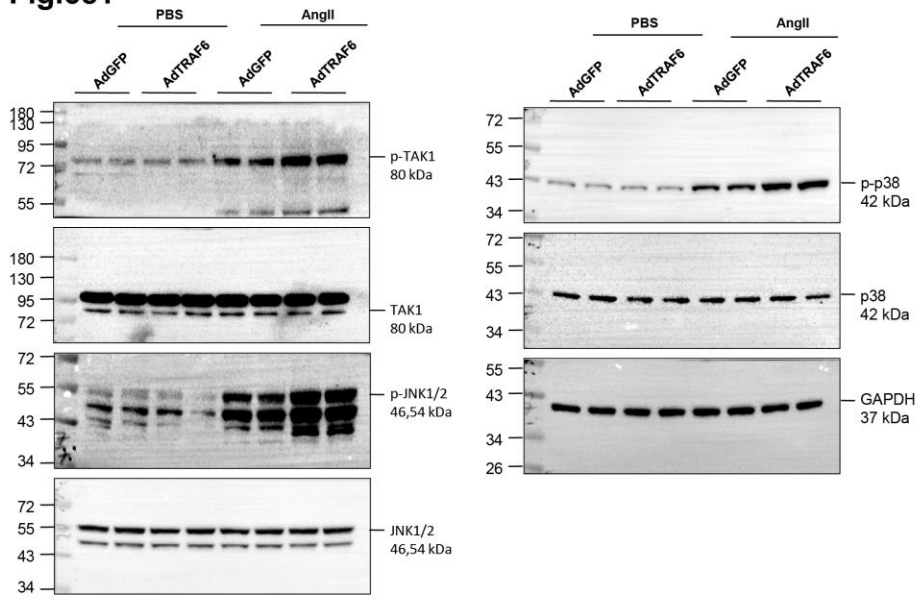

Fig.6c2

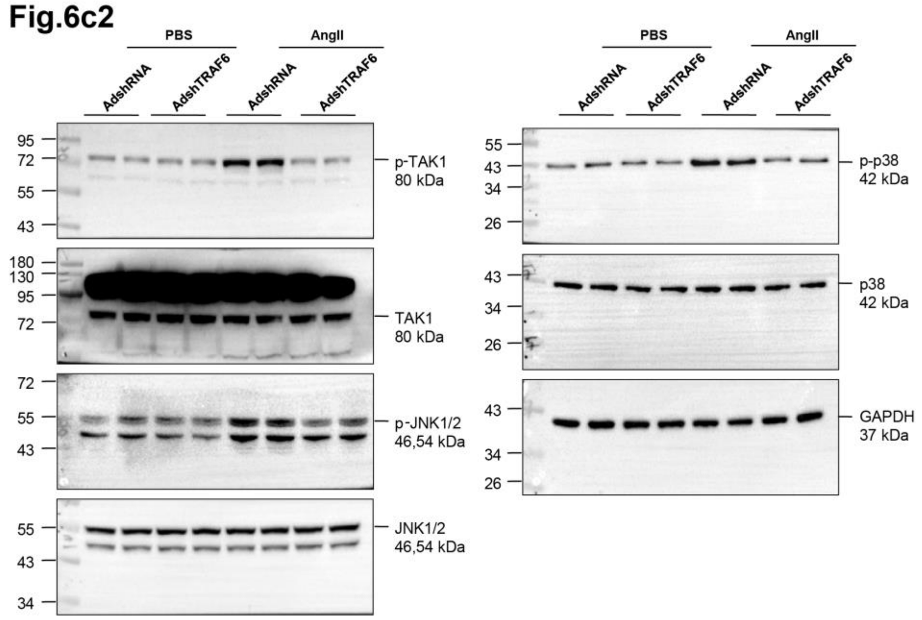

Supplementary Figure. 9. Full gel scans relating to indicated figures (continued).

Fig.6d1

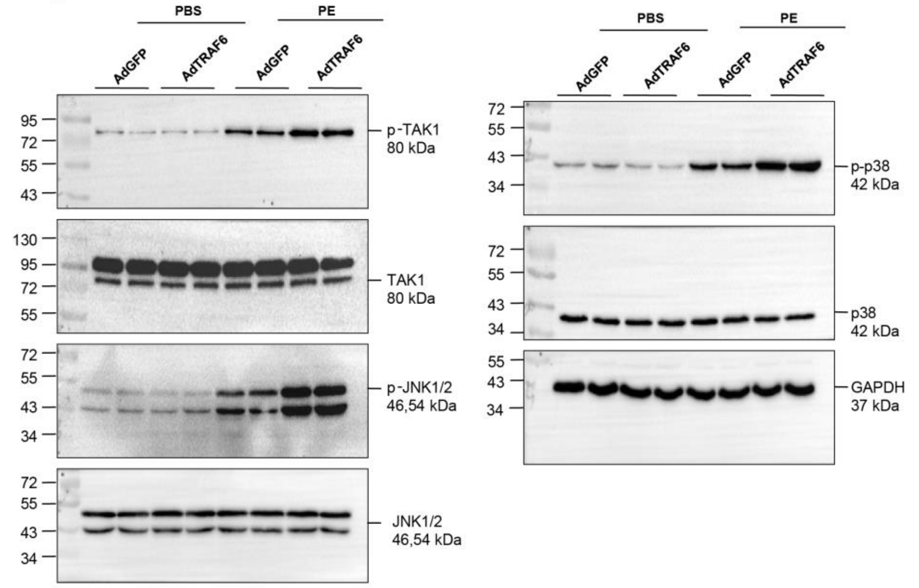

Fig.6d2

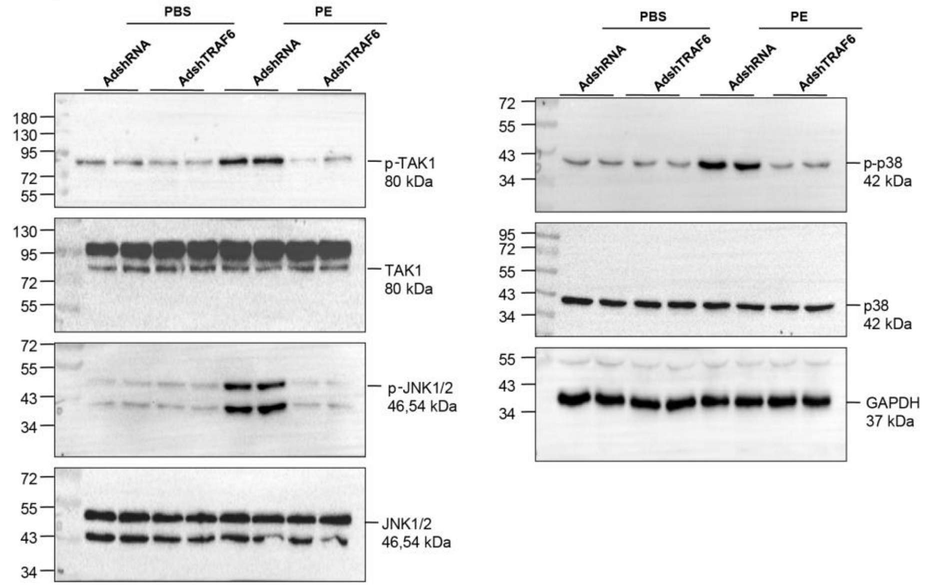

Supplementary Figure. 9. Full gel scans relating to indicated figures (continued).

Fig.6e1

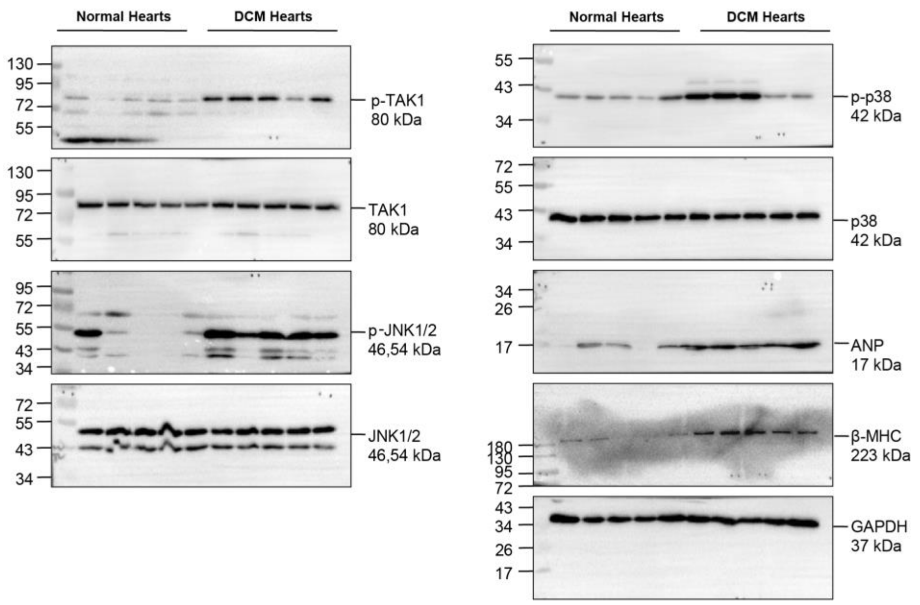

Fig.6e2

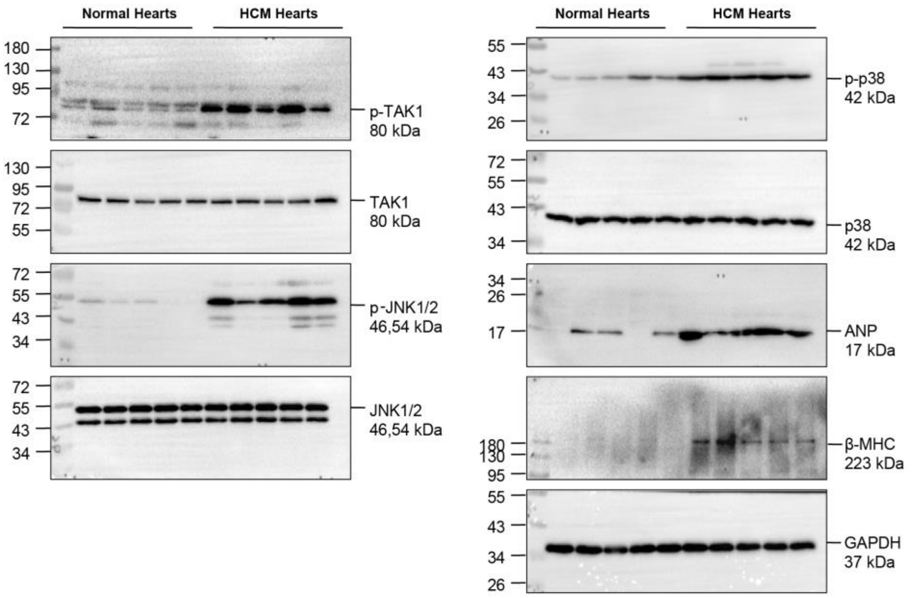

Supplementary Figure. 9. Full gel scans relating to indicated figures (continued).

Fig. 7a

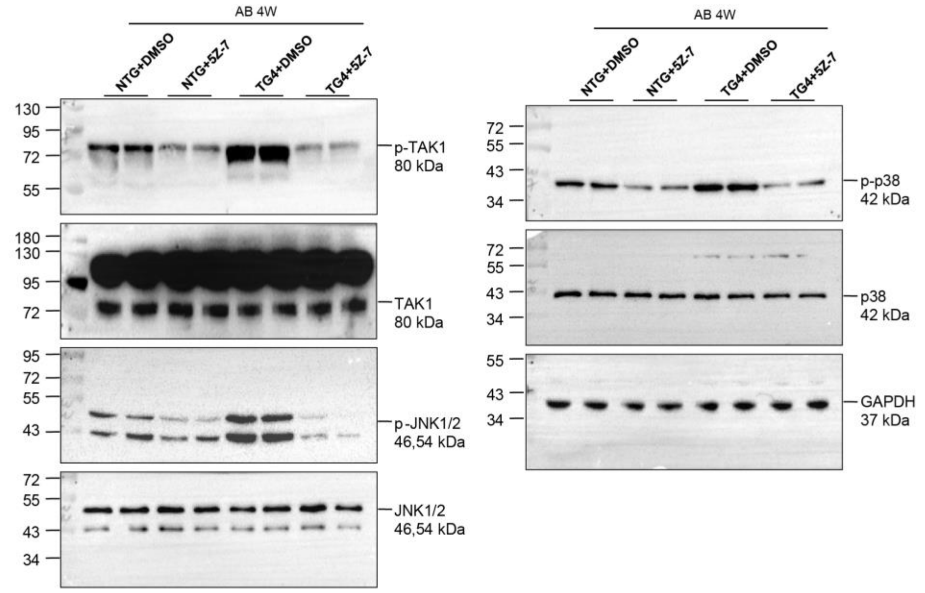

Fig.8a

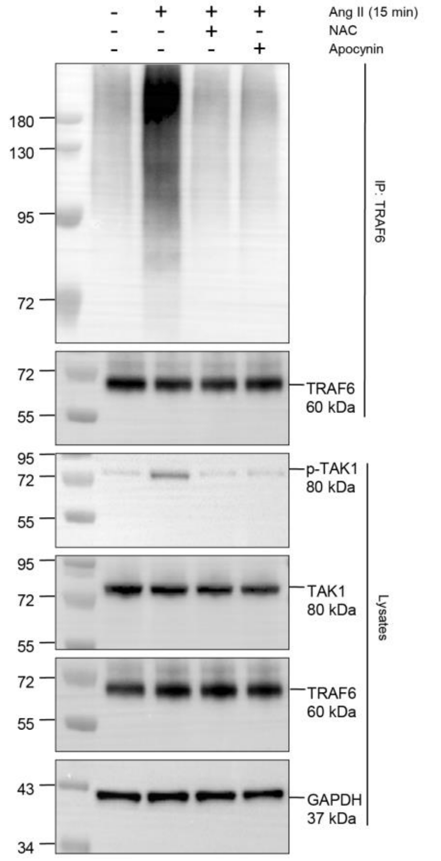

Fig.8b

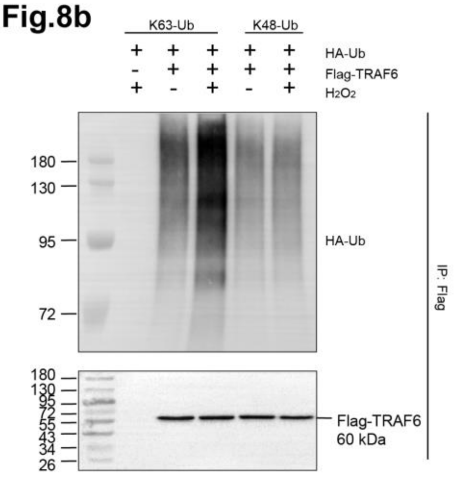

Supplementary Figure. 9. Full gel scans relating to indicated figures (continued).

Fig.8c

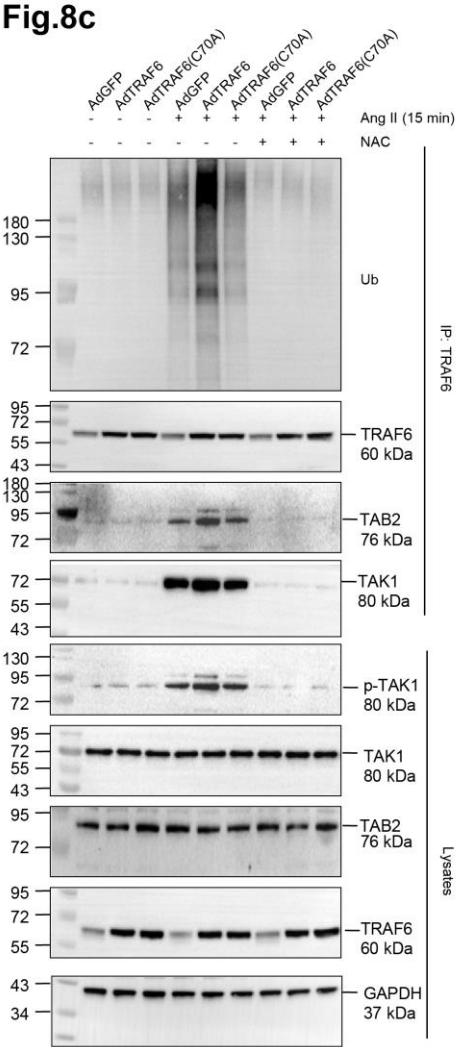

Supplementary Figure. 9. Full gel scans relating to indicated figures (continued).

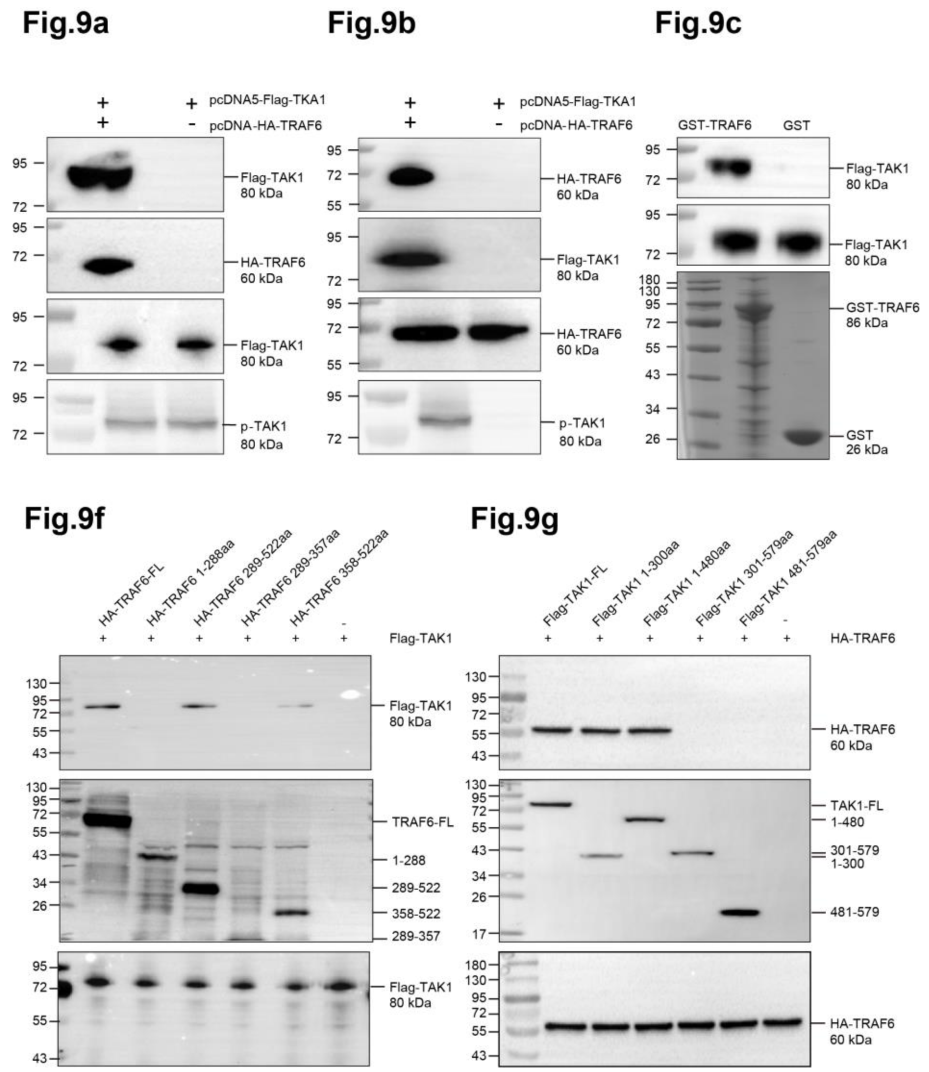

Supplementary Figure. 9. Full gel scans relating to indicated figures (continued).

Fig.9h

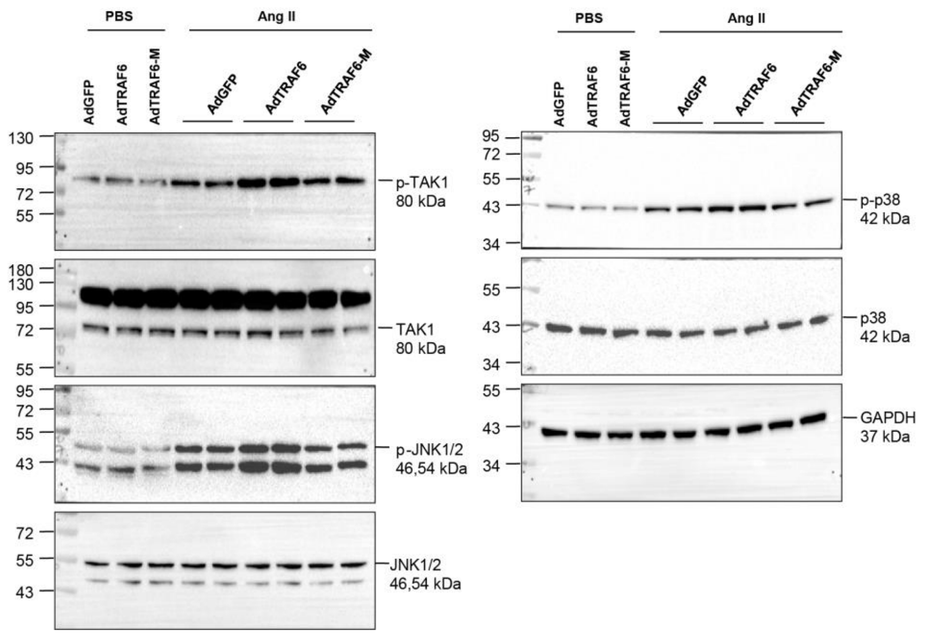

Supplementary Figure. 9. Full gel scans relating to indicated figures (continued).

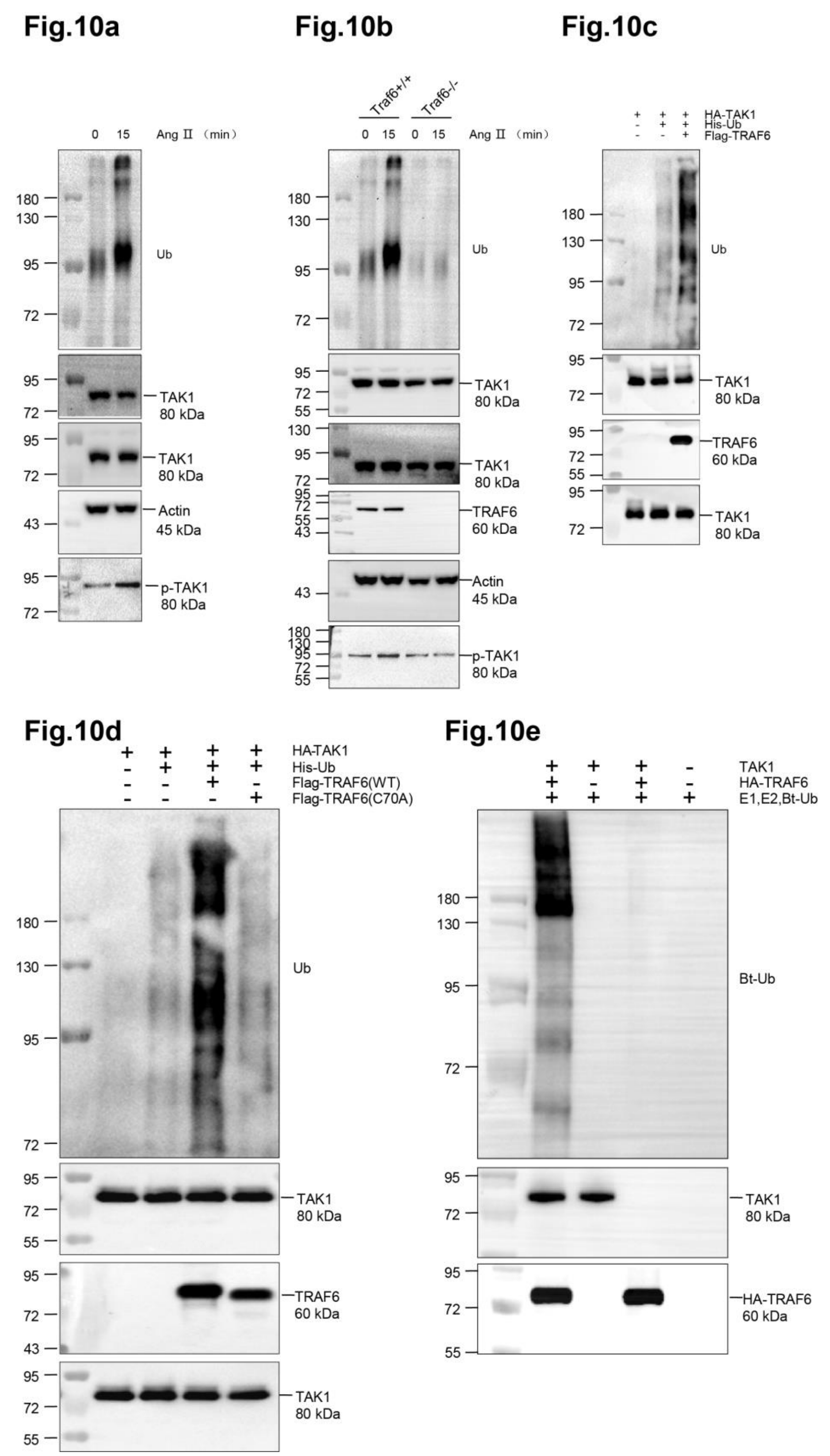

Supplementary Figure. 9. Full gel scans relating to indicated figures (continued).

Fig.S2b

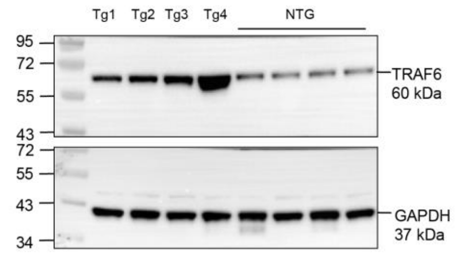

Fig.S2e1

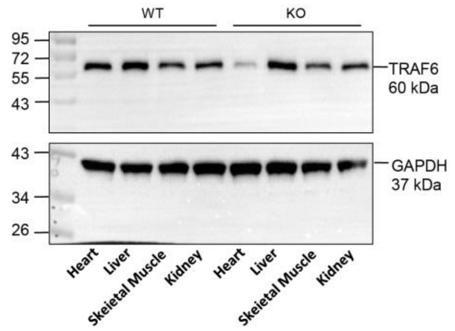

Fig.S2e2

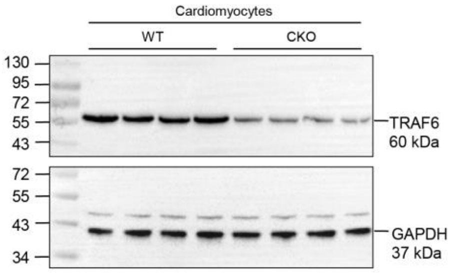

Fig.S5a1

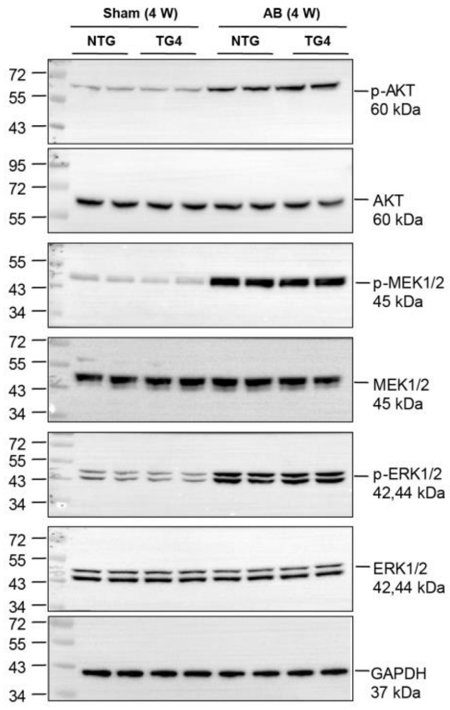

Fig.S5a2

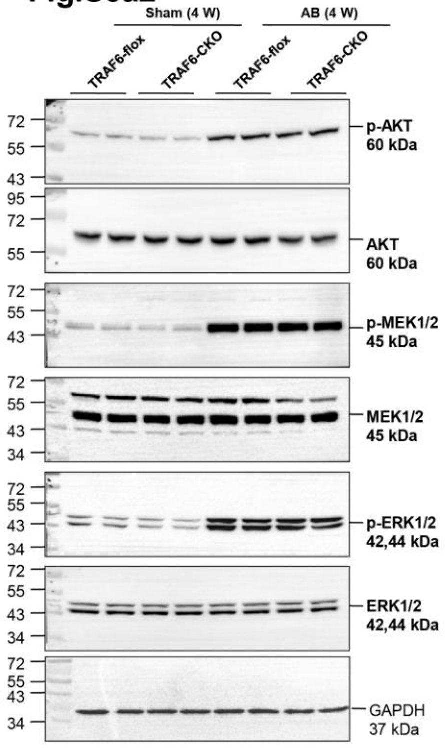

Supplementary Figure. 9. Full gel scans relating to indicated figures (continued).

Fig.S5b1

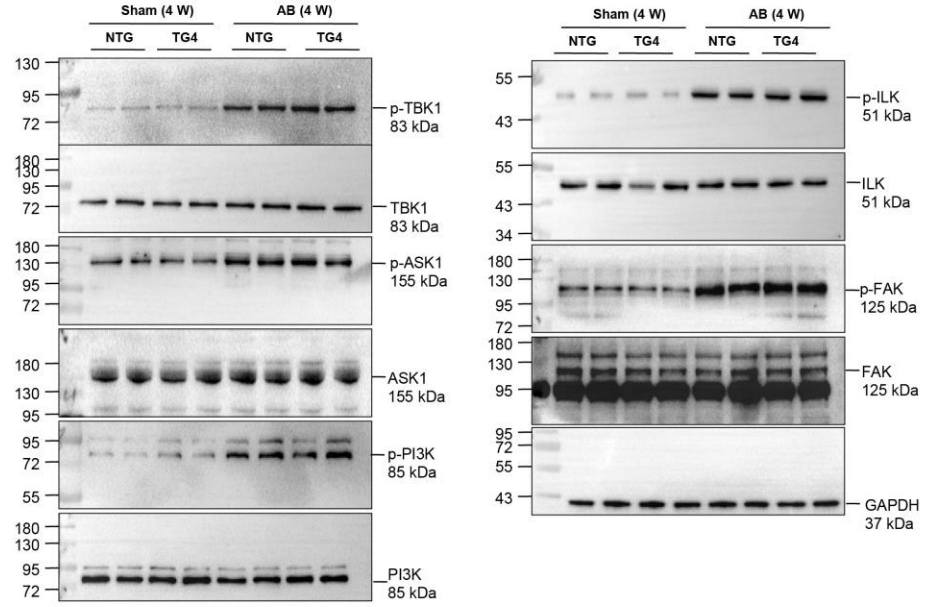

Fig.S5b2

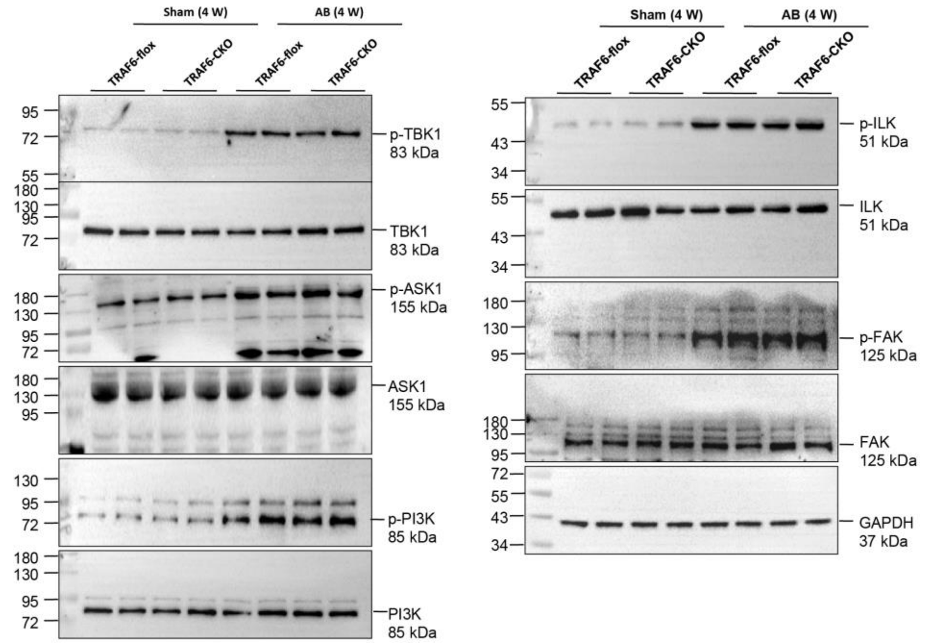

Supplementary Figure. 9. Full gel scans relating to indicated figures (continued).

Fig.S5c1

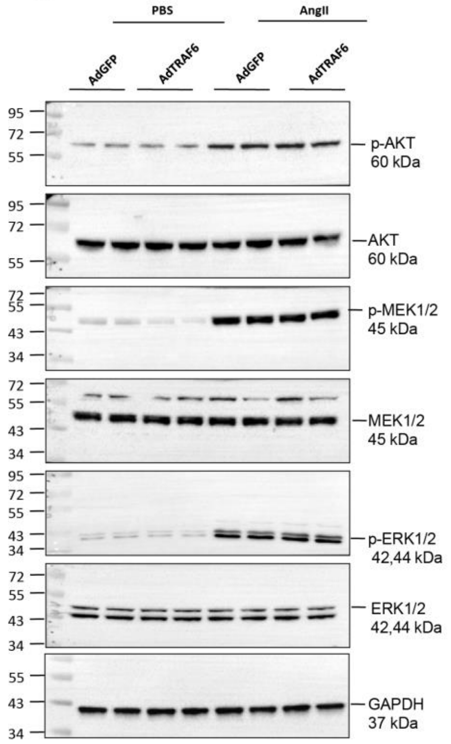

Fig.S5c2

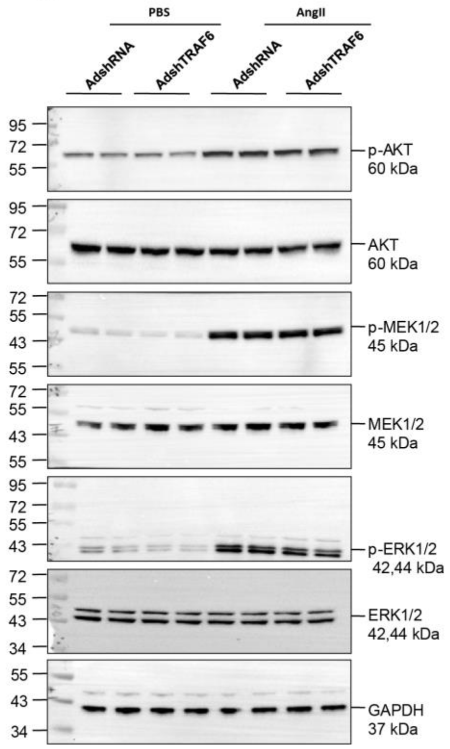

Supplementary Figure. 9. Full gel scans relating to indicated figures (continued).

Fig.S5d1

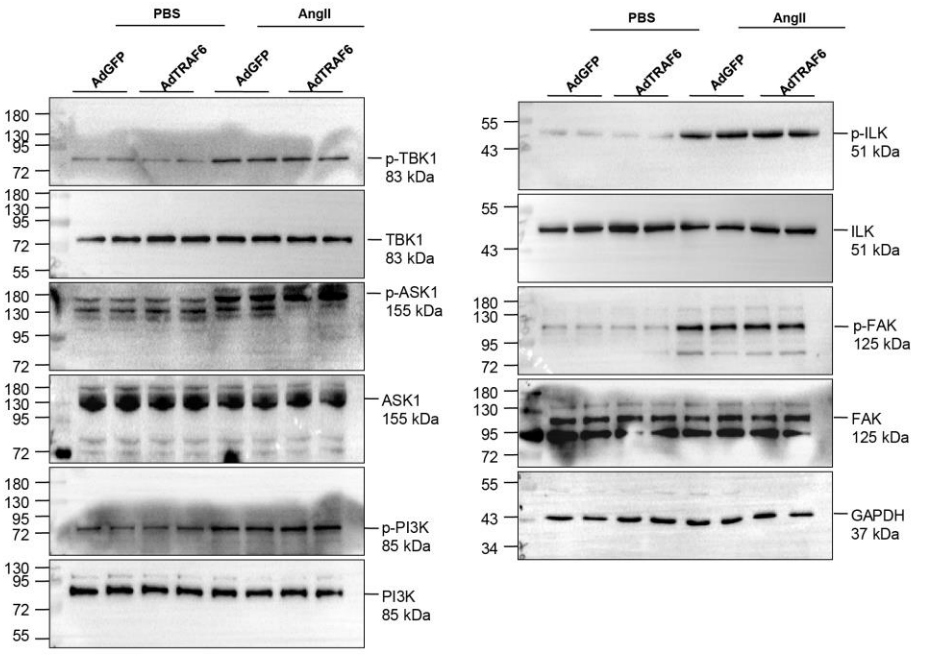

Fig.S5d2

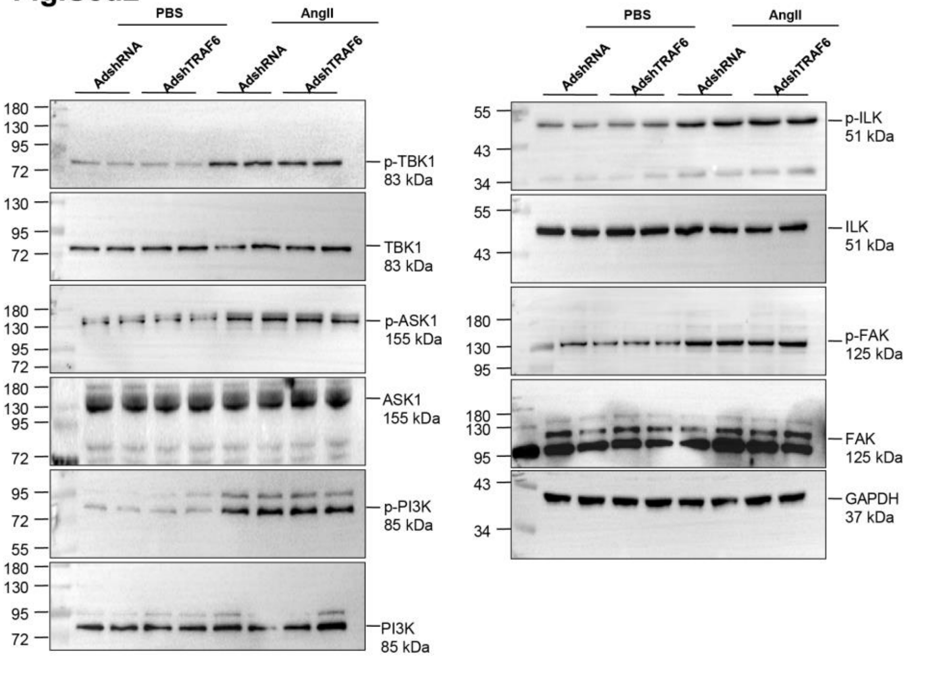

Supplementary Figure. 9. Full gel scans relating to indicated figures (continued).

Fig.S7

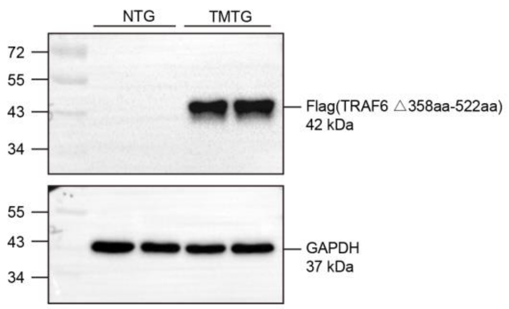

Fig.S8c

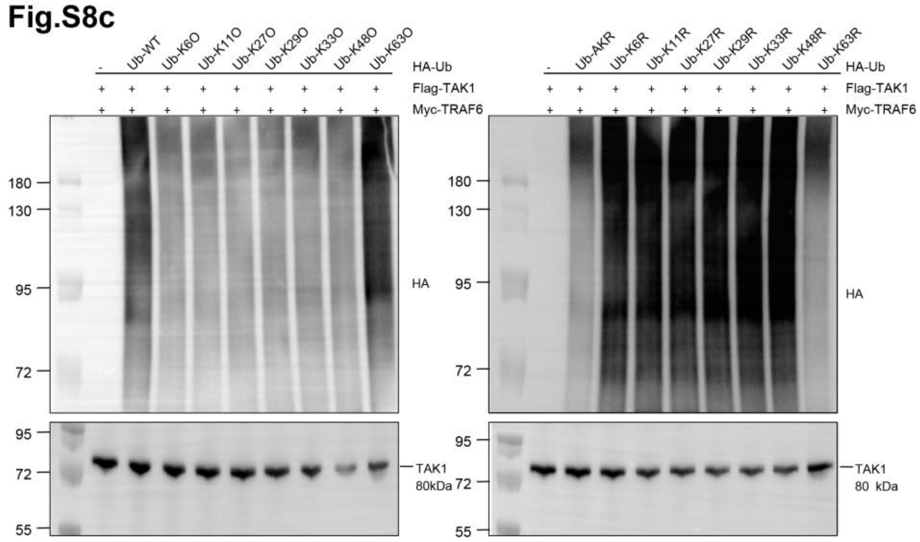

Supplementary Figure. 9. Full gel scans relating to indicated figures (continued).

Supplementary Table 1 The primers for Real-Time PCR

**Primer name**	**Forward Primer**	**Reverse Primer**
Anp-Mouse	ACCTGCTAGACCACCTGGAG	CCTTGGCTGTTATCTTCGGTACCGG
Bnp-Mouse	GAGGTCACTCCTATCCTCTGG	GCCATTTCCTCCGACTTTTCTC
β-Mhc-Mouse	CCGAGTCCCAGGTCAACAA	CTTCACGGGCACCCTTGGA
Ctgf-Mouse	TGACCCCTGCGACCCACA	TACACCGACCCACCGAAGACACAG
Collagen I-Mouse	AGGCTTCAGTGGTTTGGATG	CACCAACAGCACCATCGTTA
Collagen III-Mouse	CCCAACCCAGAGATCCCATT	GAAGCACAGGAGCAGGTGTAGA
Gapdh-Mouse	ACTTGAAGGGTGGAGCCAAA	GACTGTGGTCATGAGCCCTT

Supplementary Table 2 The primers used for plasmid construction

Primer name	Primer
TRAF6-F-BglII	GGAAGATCTATGAGTCTGCTAAACTGTGA
TRAF6-R288-XhoI	CCGCTCGAGCTAATACCCAGAGTCGGGTAT
TRAF6-F289-BglII	GGAAGATCTATCTCAGAGGTCCGGAATTTCC
TRAF6-R357-XhoI	CCGCTCGAGCTAAATCTTCCAAATATAAATTCC
TRAF6-F358-BglII	GGAAGATCTGGCAACTTTGGAATGCAT
TRAF6-R-XhoI	CCGCTCGAGCTATACCCCTGCATCAGT
TAK1-1S	CGCGGATCCATGTCTACAGCCTCTGCCGC
TAK1-301S	CGCGGATCCCCTTGTCAGTATTCAGATGA
TAK1-481S	CGCGGATCCCAGCCTCTAGCACCGTGC
TAK1-579A	CCGCTCGAGTCATGAAGTGCCTTGTCGTT
TAK1-300A	CCGCTCGAGATACTGTAATGGCTCATCTG
TAK1-480A	CCGCTCGAGTAGTTGGTGATCCAGTGTAA

Supplementary Table 3 Detailed information of human heart samples

Subject	Diagnosis	Age(years)	Gender	LVEF(%)	LVEDd(mm)	IVSd(mm)
1	Donor	57	Female	62	39	7
2	Donor	50	Female	N/A	N/A	N/A
3	Donor	28	Male	72	45	7
4	Donor	53	Male	65	47	8
5	Donor	45	Male	66	43	9
6	Donor	53	Male	63	46	9
7	DCM	63	Male	23	70	11
8	DCM	39	Female	26	60	9
9	DCM	38	Male	30	67	8
10	DCM	44	Female	22	62	10
11	DCM	56	Male	23	90	10
12	DCM	64	Male	37	80	10
13	DCM	76	Male	36	60	9
14	HCM	23	Male	57	N/A	23
15	HCM	30	Male	66	N/A	20
16	HCM	50	Male	74	N/A	31
17	HCM	40	Female	58	N/A	25
18	HCM	34	Male	61	48	22

DCM: Dilated cardiomyopathy; HCM: Hypertrophic cardiomyopathy; LVEF: Left ventricular ejection fraction; LVEDd: Left ventricular end-diastolic diameter. IVSd: Interventricular septal thickness at diastole; N/A: not available.
